# A Polymer-Derived Co(Fe)O_*x*_ Oxygen Evolution Catalyst Benefiting from the Oxidative Dehydrogenative Coupling of Cobalt Porphyrins

**DOI:** 10.1021/acscatal.3c02940

**Published:** 2023-11-08

**Authors:** Drialys Cardenas-Morcoso, Deepak Bansal, Max Heiderscheid, Jean-Nicolas Audinot, Jérôme Guillot, Nicolas D. Boscher

**Affiliations:** Materials Research and Technology Department, Luxembourg Institute of Science and Technology, 28 Avenue des Hautes-Fourneaux, Esch-sur-Alzette L-4362, Luxembourg

**Keywords:** porphyrin, conjugated polymers, oCVD, thin films, molecular transformation, oxygen evolution catalyst

## Abstract

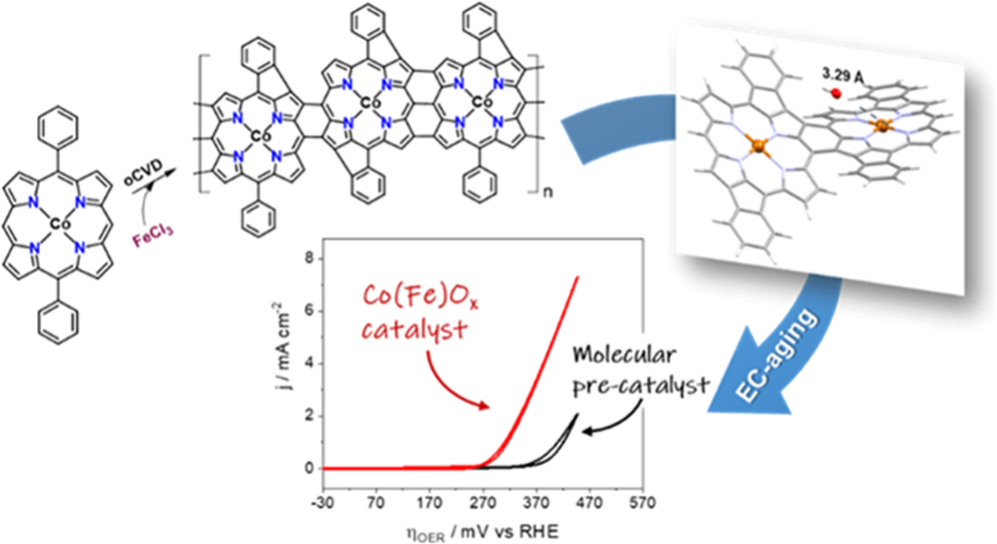

Thin films of cobalt porphyrin conjugated polymers bearing different substituents are prepared by oxidative chemical vapor deposition (oCVD) and investigated as heterogeneous electrocatalysts for the oxygen evolution reaction (OER). Interestingly, the electrocatalytic activity originates from polymer-derived, highly transparent Co(Fe)O_*x*_ species formed under operational alkaline conditions. Structural, compositional, electrical, and electrochemical characterizations reveal that the newly formed active catalyst greatly benefited from both the polymeric conformation of the porphyrin-based thin film and the inclusion of the iron-based species originating from the oCVD reaction. High-resolution mass spectrometry analyses combined with density functional theory (DFT) calculations showed that a close relationship exists between the porphyrin substituent, the extension of the π-conjugated system cobalt porphyrin conjugated polymer, and the dynamics of the polymer conversion leading to catalytically active Co(Fe)O_*x*_ species. This work evidences the precatalytic role of cobalt porphyrin conjugated polymers and uncovers the benefit of extended π-conjugation of the molecular matrix and iron inclusion on the formation and performance of the true active catalyst.

## Introduction

Molecular structures chelating first-row transition metal centers such as cobalt,^1−3^ iron,^4,5^ nickel,^6−8^ copper,^9−11^ and manganese^12,13^ are highly attractive as water oxidation catalysts, as alternatives to expensive and low-abundant ruthenium and iridium-based complexes.^14,15^ Inspired by their role in natural processes—as chlorophyll in photosynthesis—and their outstanding optoelectronic properties, metalloporphyrins and their derivatives have been intensively studied for photo- and electrocatalytic water splitting reactions, primarily as homogeneous catalysts.^16−23^ The association of porphyrins in the form of conjugated polymers can substantially improve their catalytic activity.^1,24,25^ Metal complexes of multiply-linked (triply, doubly, and singly fused) porphyrins usually exhibit enhanced physicochemical properties with respect to their monomer counterparts due to the delocalization of π-electron cloud across the entire ligand backbone.^26^ Expressly, the extended delocalization of π-electrons in porphyrin conjugated assemblies results in enhanced electric and optoelectronic properties, i.e., electronic transitions reaching the infrared region and increased conductivity,^27−29^ and improved π-*d* orbital coupling, contributing to strengthen charge transport.^30^

A major drawback of molecular complexes is the possible loss of their structural integrity upon harsh operating conditions, which often yields the formation of heterogeneous metal oxides/hydroxides acting as the real catalysts.^31,32^ Yet, the polymerization and heterogenization of molecular complexes, including metalloporphyrins, stands as a practical strategy to increase both their catalytic activity and durability.^33−35^ Recently, our group reported directly fused nickel(II) porphyrins deposited on fluorine-doped tin oxide (FTO) coated glass as heterogeneous single-site catalysts for the oxygen evolution reaction (OER), showing higher activity and stability than their monomeric counterparts in alkaline conditions.^25^

In this context, cobalt porphyrins have been actively investigated as single-site OER catalysts.^16,36^ However, only a few studies refer to their integration as heterogeneous catalysts.^37−39^ For instance, Han and co-workers reported the investigation of cobalt porphyrin thin films coated on FTO as heterogeneous catalysts for water oxidation, achieving faradaic efficiencies close to 100%.^37^ It was later revealed that cobalt porphyrins deposited on FTO promptly decompose into a thin film of highly active CoO_*x*_ on the electrode surface during water oxidation.^38^ More recently, Zhan and co-workers reported that the electropolymerization of cobalt porphyrins and corroles on carbon cloth electrodes for OER results in improved OER performance and high stability in comparison to their simple immobilization on conductive substrates.^40^ Although existing reports evidenced the conversion of cobalt coordination compounds into inorganic catalysts (generally cobalt oxide species) under water oxidation operational conditions,^41−44^ the detailed structural characterization of the formed species is often highly challenging with conventional EDS, XRD, or XPS analysis.^38^

On the other hand, heterogeneous transition (bi)metal catalysts frequently outperform single metal catalysts toward the OER in alkaline media.^45−48^ Benchmarking protocols have placed nickel–iron oxide (NiFeO_*x*_) and cobalt–iron oxide (CoFeO_*x*_) among the most active OER catalysts in alkaline solutions, particularly at lower overpotentials.^49,50^ Previous investigations showed that even the incorporation of trace amounts of iron (e.g., from nonpurified solutions used as electrolytes) significantly enhances the activity of pure NiO_*x*_^51,52^ and CoO_*x*_.^53^ However, the role of iron inclusion as an activity mediator or actual active site is in active debate in the electrocatalysis community.^54,55^ For example, based on in situ mass and conductivity measurements, Burke and coauthors showed that, similarly to NiOOH, CoOOH serves primarily as a conductive and chemically stable host that enhances the activity of active Fe sites.^53^ Conversely, from theoretical calculations, the local configuration of Fe(III) was found to improve the activity of CoOOH, providing optimal absorption energy of OER intermediates (Δ*G*_OH_ and Δ*G*_O_ – Δ*G*_OH_) at the Co site due to the difference in electron affinity between Co(IV) and Fe(III).^56^

Recently, our group implemented the oxidative chemical vapor deposition (oCVD) strategy for the straightforward preparation of fused-metalloporphyrins conjugated polymer thin films over a wide variety of substrates.^57−61^ In oCVD, iron(III) chloride (FeCl_3_) is used as a suitable volatile oxidant to promote the dehydrogenative coupling reaction between porphyrins monomers possessing free *meso-*positions.^57,59^ Although the inclusion of both unreacted oxidant and oxidant byproducts disseminated inside the oCVD thin films have been previously acknowledged,^58,62^ the possible impact of such iron-containing residuals on the catalytic performances of the fused metalloporphyrin conjugated polymers has not been elucidated.

**Scheme 1:**
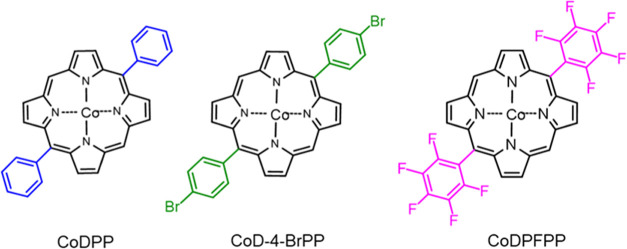
Molecular Structure of the 5,15 Di-substituted Cobalt(II) Porphyrins Investigated

Herein, we report the simultaneous synthesis and deposition of fused cobalt porphyrin conjugated polymers thin films via oCVD onto FTO substrates and its role as a precatalytic polymeric matrix for the formation of the OER active electrocatalyst species, Co(Fe)O_*x*_. We show that the in situ electrochemical conversion of fused cobalt porphyrin conjugated polymers in alkaline conditions benefits from both the precatalytic polymeric structure and the presence of residual Fe-containing compounds originating from the oCVD reaction. Three cobalt(II) 5,15-diaryl porphyrins bearing different *meso*-substituent, i.e., cobalt(II) 5,15-diphenyl porphyrin (CoDPP), cobalt(II) 5,15-di-4-bromophenyl porphyrin (CoD-4-BrPP), and cobalt(II) 5,15-dipentafluorophenyl porphyrin (CoDPFPP), represented in Scheme 1, were used to study the polymer conversion. The substituents were selected based on previous reports on the successful integration of cobalt(II) 5,10,15,20-tetra-aryl porphyrins for electrocatalytic water oxidation^37^ and in perspective to elucidate the impact of the fused cobalt porphyrin conjugated polymer constitution—occurrence or prevention of intramolecular cyclization side reaction^58,60^—on the true catalyst formation and final activity.

## Results and Discussion

### Fused Cobalt Porphyrin Conjugated Polymer Thin Films for OER: Molecular Catalyst or Precatalytic Matrix?

**Figure 1:**
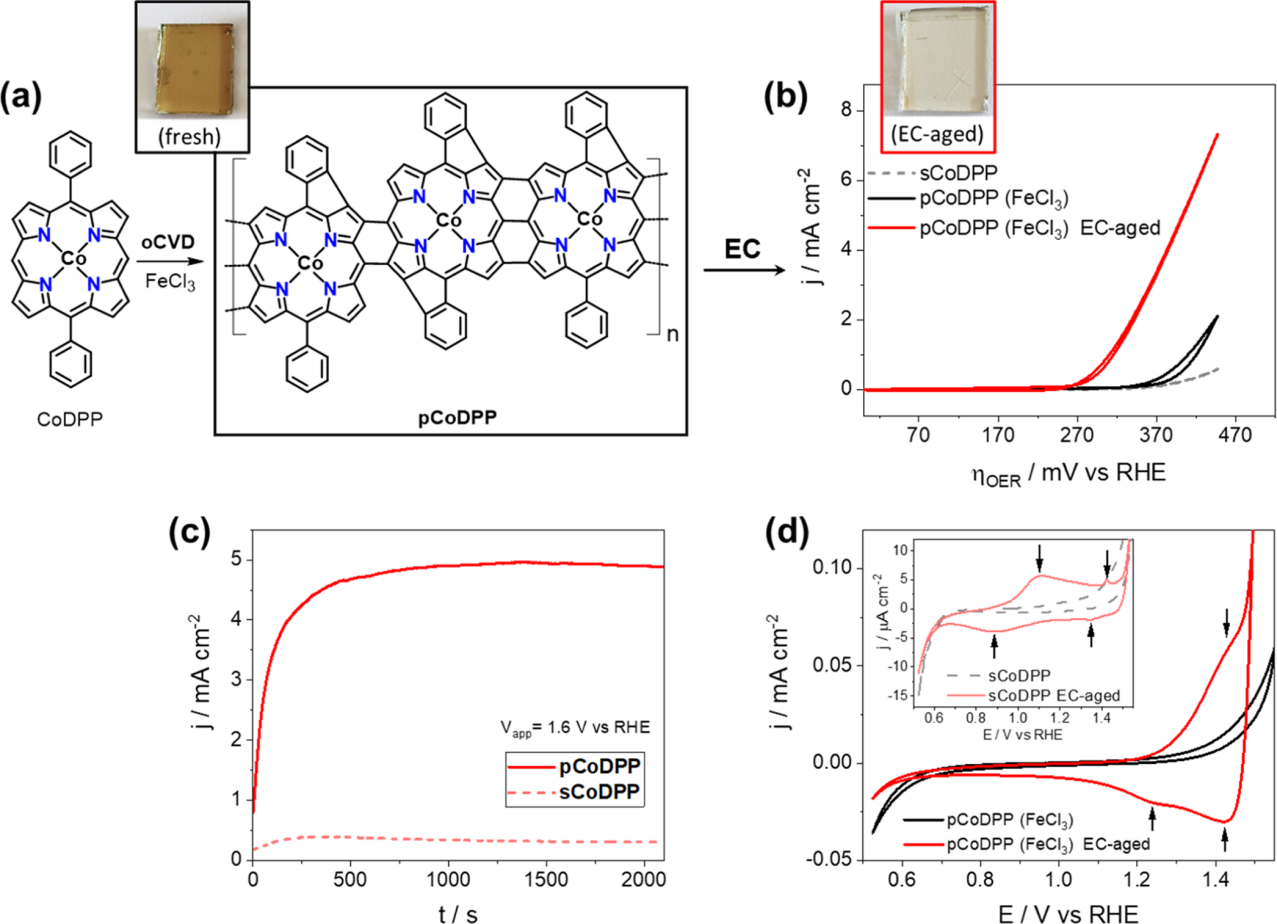
(a) Representation of the oCVD process to form directly fused cobalt porphyrin conjugated polymeric chains from 5,15-Co(II) diphenyl porphyrin (CoDPP) in the presence of FeCl_3_. The digital picture of the as-prepared **pCoDPP** thin film on FTO is shown as an inset. (b) CVs of the as-prepared **sCoDPP** (sublimed monomer) and **pCoDPP** thin films, and **pCoDPP** after EC-aging. The digital picture of the **pCoDPP** electrode after EC-aging is shown as an inset. (c) Chronoamperogram showing the early current increase of **pCoDPP** electrodes in comparison with **sCoDPP** during EC-aging. (d) Zoomed region of the CV of the **sCoDPP** (inset) and **pCoDPP** electrodes before and after EC-aging.

Our study started with the investigation of directly fused cobalt(II) 5,15-diphenyl porphyrin conjugated polymer thin films (**pCoDPP**), based on the previous superior OER properties of cobalt(II) porphyrin substituted with phenyl groups.^36,37^ All of the details regarding the deposition conditions (description of the oCVD reactor, amount, and sublimation temperatures for the monomer and oxidant) for both the conjugated polymer thin films prepared by oCVD (**pCoDPP**) and reference monomer thin films prepared by sublimation (**sCoDPP**) are provided in the Supporting Information, Scheme S1 and Table S1. Figure 1a represents the dehydrogenative intermolecular coupling (polymerization) reaction of CoDPP in the presence of FeCl_3_ to form fused cobalt porphyrin conjugated polymer thin films. One should note that intramolecular dehydrogenative coupling can also occur between the free *ortho-*position of the aryl substituent and *β*-positions of the porphyrin macrocycle, along with the intermolecular coupling reaction. The inset in Figure 1a shows the intense greenish coloration of **pCoDPP** on FTO coherent with the formation of directly fused porphyrin conjugated polymers.^57−60^ Consistently, laser desorption/ionization high-resolution mass spectrometry (LDI-HRMS) analysis of **pCoDPP** showed peak distributions in the LDI-HRMS spectra related to oligomers [(CoDPP)*_n_* – (H_2_)*_m_*]^+^ with *m* ≥ *n*, indicating the successful polymerization of the porphyrin units (Figure S1, Supporting Information). Besides intermolecular dehydrogenative coupling, the elimination of more than three 2H pairs for the CoDPP dimer (Figure S1) indicates the occurrence of an intramolecular dehydrogenative coupling between the phenyl substituent and the porphyrin core. Other side reactions, such as chlorination of the macrocycle or/and the phenyl substituent and oxidation of the central metal cation, can also occur in oCVD when using FeCl_3_ as an oxidant.^58−60,62^ As depicted in Figure S1, chlorination is evident in **pCoDPP**, giving rise to peak distributions shifted from 35 *m*/*z*, corresponding to the exchange of hydrogen by chlorine atoms. LDI-HRMS revealed the inclusion of oxygen in both the conjugated polymer thin films prepared by oCVD and the sublimed monomer. Notably, the LDI/HRMS spectrum of **sCoDPP** also displays the presence of oxygen inclusion, pointing to oxidation of the porphyrin that occurs not only during the oCVD process but also under ambient exposure conditions.

The as-prepared **pCoDPP** and **sCoDPP** electrodes were initially tested by cyclic voltammetry (CV) in alkaline electrolyte (1 M KOH, pH 13.6) (see the Supporting Information, Experimental and theoretical methods for further details). As depicted in Figure 1b (gray dashed line), the as-prepared sublimed porphyrin thin films, **sCoDPP**, showed a moderate OER activity with a 370 mV onset overpotential to OER (η_OER_). Nevertheless, these values are comparable with previous reports on cobalt porphyrins for OER in similar testing conditions.^36^ The electrocatalytic activity of the **pCoDPP** thin film (black straight line) is slightly enhanced compared to the one of the sublimed **sCoDPP** thin films, with a 350 mV η_OER_ and a 4-fold enhancement of the current density at 1.65 V vs RHE. These observations confirm the significance of the intermolecular dehydrogenative coupling reaction (polymerization) to enhance the electrocatalytic activity of metalloporphyrins.^25^ The **pCoDPP** and **sCoDPP** electrodes were further electrochemically treated by applying a constant potential of 1.6 V vs RHE for 2 h (hereafter, “EC-aging” refers to this treatment). As shown in Figure 1c, the chronoamperogram for the as-deposited **pCoDPP** thin film depicts an early and rapid increase of the current density close to 5 mA cm^–2^, presumably related to the transformation of the initial **pCoDPP** thin film into cobalt oxide species (CoO_*x*_) according to previously reported in situ electrochemical conversion of cobalt porphyrins immobilized on FTO substrates.^38^ After EC-aging, a notable change in the electrode surface appearance was observed, from the intense greenish coloration of the as-prepared **pCoDPP** thin film (Figure 1a) to a fully transparent electrode, as shown in the inset of Figure 1b. The modified **pCoDPP** electrode, which was retested by CV, exhibited a significantly improved performance (Figure 1b, red solid line) with a notable η_OER_ shift to 260 mV and an increased current density in agreement with the behavior observed during EC-aging (Figure 1c). Such observations point toward the formation of new species acting as true catalysts for OER, as reported in other cobalt-based complexes.^38,41,42^ Nonetheless, in contrast to the oCVD **pCoDPP** thin film, the reference **sCoDPP** thin film (sublimed monomer) displays a reduced η_*OER*_ shift and a less significant current density increase after EC-aging (Figure S2), suggesting the positive role of the oCVD reaction in the transformation of the molecular precatalyst into OER active cobalt oxides species.

Furthermore, the EC-aged **pCoDPP** electrodes were rinsed with acetone to remove possible organic residuals resulting from the polymer degradation during electrochemical aging and retested by CV. As shown in Figure S3, the OER onset potential remains unaltered while the current density slightly increases, pointing to the origin of the catalytic activity on the newly formed species at the FTO surface rather than the former porphyrin conjugated polymer.^38^ Beyond, redox features not observed initially on the as-prepared **pCoDPP** and **sCoDPP** thin films were revealed after EC-aging, as depicted in Figure 1d. Specifically, the EC-aged **sCoDPP** electrode (sublimed monomer) showed a redox peak with *E*_1/2_ ca. 1.0 V vs RHE. Such a feature, previously observed by Daniel and co-workers on spin-coated cobalt porphyrin on FTO electrodes after 20 CV scans,^38^ is ascribed to cobalt species transformation under alkaline OER conditions, in particular, the Co(II) to Co(III) oxidation about 1.12 V vs RHE in the forward scan.^44,63,64^ A second redox feature observed at *E*_1/2_ ca. 1.38 V vs RHE is assigned to the subsequent Co(III) oxidation to Co(IV) at ca. 1.42 V vs RHE in the forward scan.^63,64^ Conversely, the CV of the EC-aged **pCoDPP** thin film show positively shifted anodic and cathodic peaks with larger integrated area, in particular, for the redox feature corresponding to Co(IV)/Co(III), and the complete suppression of the precatalysis Co(II) to Co(III) oxidation peaks (Figure 1d). These features are attributed to the irreversible oxidation of Co(II) to Co(III) species, namely, CoOOH and Co_3_O_4_, followed by reversible oxidation to CoO_2_.^53,64−66^ Consistently with previous report on cobalt(iron) (oxy)hydroxide water oxidation catalysts, the anodic shift of these redox features points to a strong electronic interaction between cobalt and iron, which alters the electronic structure of the catalyst limiting the oxidation of Co(II) species with iron inclusion.^53^ It is worth noting that no redox features associated with iron species are observed in the potential range investigated for the electrochemical measurements, thus remaining as Fe(III).^53,67^ The above observations point to a stabilization of higher oxidation states on the Co(Fe)O_*x*_ phase resulting from the EC-aging of **pCoDPP** thin films, favoring the OER catalysis fostered by the inclusion of Fe(III).^63,66^*In situ*/*operando* investigations reporting the development of Co-based catalysts under alkaline^64^ and neutral^68^ conditions suggested Co(IV) to be a key intermediate for OER catalysis. However, recent studies on cobalt oxide^69,70^ and cobalt (oxy)hydroxide^71^ species revealed that Co(III)–OH sites play a significant role in improved catalytic activity, allowing faster OER kinetics than Co(IV)=O sites. Moreover, the modulated electronic structure resulting from the inclusion of iron(III) in Co-based catalysts appears to retain Co(III)–OH active sites and maintain an intermediate-spin state, resulting in efficient and robust OER electrocatalysis.^63^

**Figure 2:**
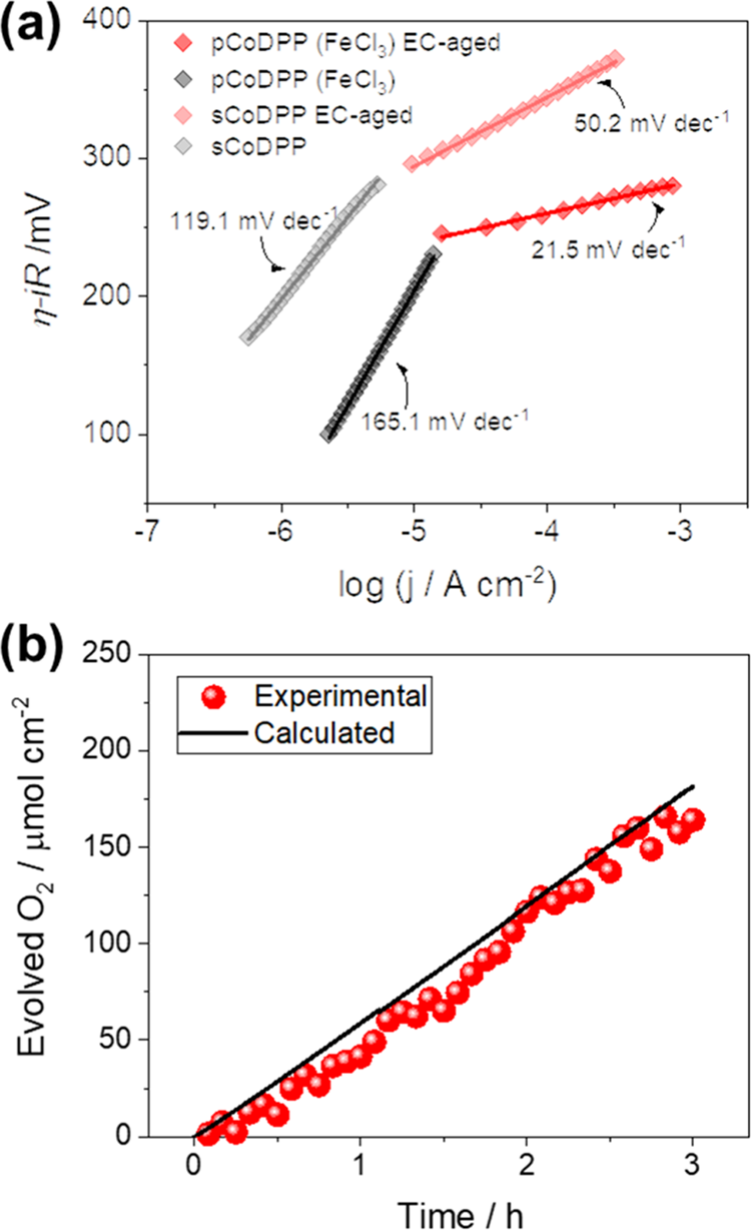
(a) Tafel plot obtained from the *iR* corrected CVs of the **sCoDPP** and **pCoDPP** electrodes before and after EC-aging. (b) Evolved O_2_ amount at the EC-aged **pCoDPP** electrode surface and theoretical amount derived from the Faraday’s law.

Figure 2a shows the Tafel plots calculated from the CVs performed on the **sCoDPP** and **pCoDPP** electrodes before and after EC-aging. Interestingly, after EC-aging and henceforth upon the formation of the new catalytically active species, the relatively large Tafel slopes of both **sCoDPP** and **pCoDPP** electrodes significantly decrease, leading to a remarkable 21.5 mV dec^–1^ value for the EC-aged **pCoDPP** electrode. Such a low Tafel slope is consistent with previous investigations on cobalt and nickel OER catalysts, where moderate iron content yielded Tafel slopes of 25–40 mV dec^–1^, while pure cobalt films exhibit Tafel slopes of ca. 62 mV dec^–1^.^63,66^ Noteworthy, while a Tafel slope near 60 mV dec^–1^ is associated with a rate-limiting chemical step before the first electron transfer according to the classical four electron/proton-transfer mechanism, a value near 24 mV dec^–1^ implies the third electron transfer as the rate-limiting step,^53^ evidencing the influence of iron inclusion in the OER mechanism, and further increase of the intrinsic catalytic activity of Co(Fe)O_*x*_ species.

To further verify the crucial role of iron inclusion from the oxidant used during the oCVD on the electrochemical transformation of **pCoDPP** thin films and the final OER activity of the polymer-derived catalyst, an iron-free oxidant, copper(II) chloride (CuCl_2_),^59^ was used as an alternative to ensure the oCVD reaction of CoDPP. Figures S4a,b of the Supporting Information provide the detailed structural and compositional characterization of the resulting thin film, confirming the formation of a directly fused porphyrin conjugated polymer. The as-prepared **pCoDPP (CuCl**_**2**_**)** thin film showed a slightly higher performance than that of the as-prepared sublimed **sCoDPP** thin film and a similar η_*OER*_ onset than **pCoDPP** prepared from FeCl_3_, yet with lower current density at higher potentials (Figure S5a). After EC-aging (Figure S5b), **pCoDPP (CuCl**_**2**_**)** showed a slightly increased current density and catholically shifted η_OER_ onset compared to the as-prepared **pCoDPP (CuCl**_**2**_**)** thin film. Specifically, after EC-aging, both the **pCoDPP** thin films prepared from FeCl_3_ and CuCl_2_ exhibit an η_OER_ of ca. 260–270 mV, while EC-aged **sCoDPP** shows a 320 mV η_OER_ (Figure S2). These observations highlight the positive role of polymerization in the formation of porphyrin-derived cobalt oxide catalysts.

The slightly improved OER performance (compared with the monomer) of the EC-aged **pCoDPP** thin film prepared using CuCl_2_ may be attributed to the formation of copper oxides and mixed copper–cobalt oxides, which have also been reported as promising OER catalysts.^72^ However, one should note that the catalytic activity of these compounds appears highly dependent on the synthetic method conditioning key properties such as crystallography structure, surface morphology, and mixed cationic valence.^72−74^ For instance, copper–cobalt oxide electrocatalyst (CCO) synthesized via coprecipitation method and thermal oxidation treatment showed the best activity and durability for a Cu/Co ratio of 1:2.^75^ Alternatively, the excellent OER activity and stability of nanowires aggregated urchin-like cobalt–copper (hydr)oxide synthesized via electrochemical transformation of cobalt–copper sulfides and attributed to the 3D nanostructure generated during the anion exchange and structure reconstruction process.^76^ Notably, the iron inclusion from FeCl_3_ on electrochemically reconstructed Co-based catalyst in alkaline conditions was recently reported to afford optimal electronic structure and energy of the OER intermediates, resulting in excellent catalytic activity and stability.^63^ As shown in Figure S5, the OER kinetics of the EC-aged **pCoDPP (CuCl**_**2**_**)** electrode persist sluggish, as current density remains far below those depicted by the EC-aged **pCoDPP** thin films prepared from FeCl_3,_ containing residual iron species prone to interact with cobalt centers during the porphyrin conjugated polymer transformation. Therefore, the improved activity of the iron-containing EC-aged **pCoDPP** thin film with respect to both the monomer and copper-containing polymer counterparts confirms the synergy between the polymeric conformation of the molecular precatalyst, improving the reactivity of porphyrin conjugated polymers, and the presence of iron originating from the oxidant used in the oCVD, whose strong coupling interaction with cobalt leads to enhanced OER activity.

The oxygen evolution at the EC-aged **pCoDPP** electrode was quantitatively assessed by gas chromatography (GC) measurements at an overpotential of 370 mV, i.e., 1.6 V vs RHE of constant applied potential. The expected O_2_ amount was calculated from the recorded chronoamperogram using the Faraday’s law (Figure S6a). The faradaic efficiency (FE) was obtained from the ratio between the experimental amount of evolved O_2_ detected by GC and the theoretical value from the measured current density (see the Experimental Section in the Supporting Information for details). As shown in Figures 2b and S6b, O_2_ is produced at the EC-aged **pCoDPP** surface electrode with 90% faradaic efficiency after 3 h of operation, with a maximum evolution rate of 60 μmol·cm^–2^ h^–1^.

**Figure 3:**
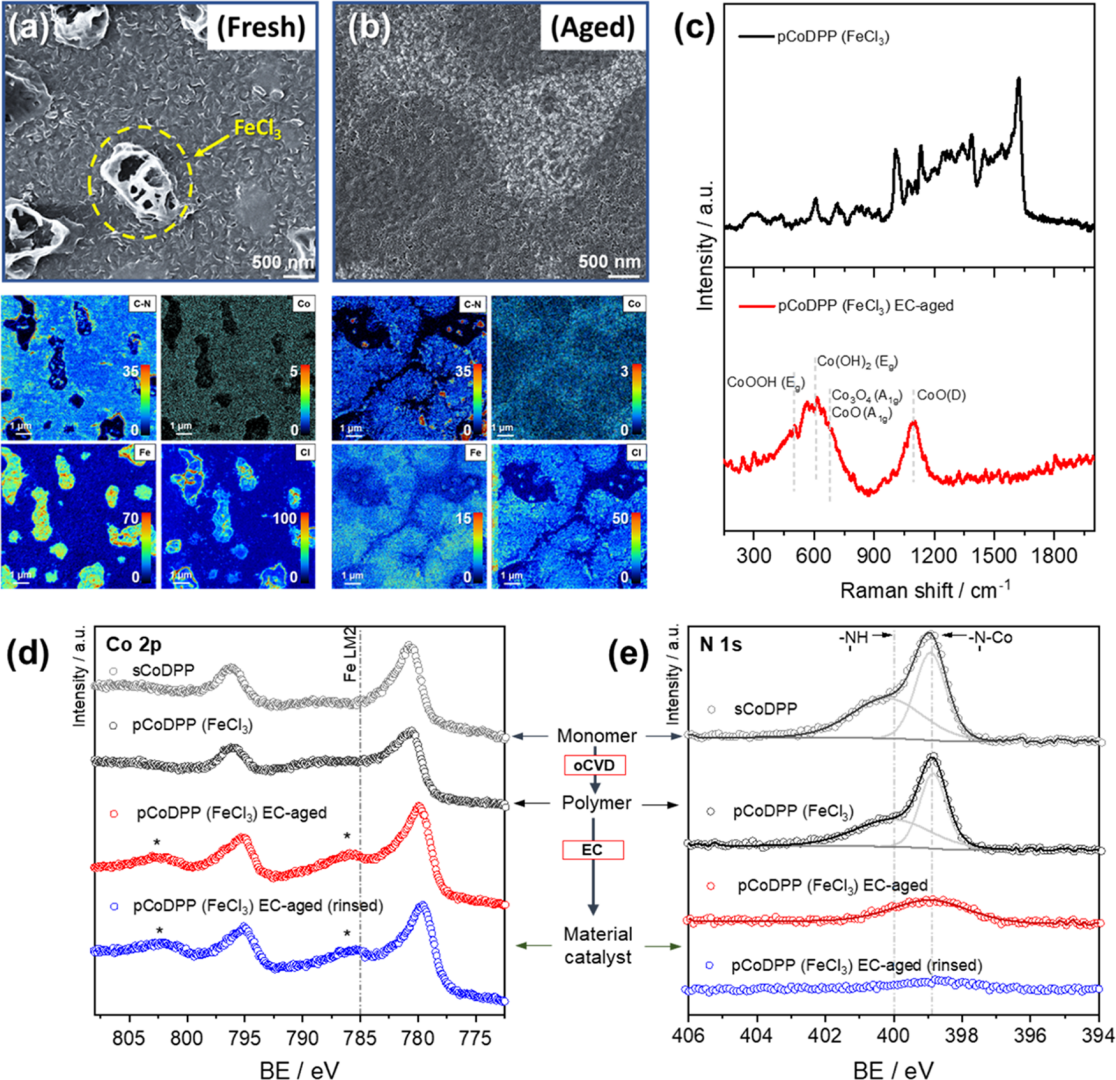
(a) Secondary electron (SE) image and elemental mapping obtained by HIM-SIMS analysis of the as-prepared **pCoDPP** thin film surface, and (b) after EC-aging. (c) Raman spectra of the as-prepared (top) and EC-aged (bottom) **pCoDPP** thin films. The Raman modes of the cobalt species are indicated for reference. (d) XPS spectra of the Co 2p and (e) N 1s core levels of, from top to bottom: the reference **sCoDPP** sublimed thin film, the as-prepared **pCoDPP** conjugated polymer, **pCoDPP** after EC-aging, and EC-aged **pCoDPP** thin film rinsed with acetone.

Structural and compositional characterization was carried out in both the as-prepared and EC-aged **pCoDPP** (prepared using FeCl_3_) and reference sublimed **sCoDPP** electrodes to identify the newly formed active catalytic species and better understand the influence of the oCVD reaction on the porphyrin transformation to an efficient OER catalyst. The morphology and chemical elements distribution at the electrode surfaces were evaluated by Helium Ion Microscopy coupled with Secondary Ion Mass Spectrometry (HIM-SIMS) analysis. Figure 3a shows the secondary electron (SE) image of the as-prepared **pCoDPP** electrode, characterized by the presence of FeCl_*x*_ clusters remaining from the oCVD reaction, as confirmed by the intense Fe and Cl signals of the elemental mapping analysis. The C–N and Co chemical mapping provides evidence of the uniform distribution of the cobalt porphyrin conjugated polymer across the electrode surface. In contrast, the surface morphology of the electrode significantly changes after EC-aging, as depicted in Figure 3b, with evident damage of the organic moiety, as depicted in the C–N mapping region, with an apparent agglomeration of cobalt. Interestingly, the elemental mapping corresponding to Fe confirms that iron-containing species remain at the electrode surface, not only in the residual organic phase but also in the cobalt-rich regions, suggesting the formation of mixed cobalt–iron oxide species, i.e., Co(Fe)O_*x*_.

Comparison of the Raman spectrum of the as-prepared and EC-aged **pCoDPP** thin film (Figure 3c) evidenced the transformation of the initial cobalt porphyrin conjugated polymer into mixed cobalt oxides species. Expressly, the Raman spectrum of the EC-aged **pCoDPP** electrode showed a broad signal centered at 607 cm^–1^ and assigned to the E_g_ Raman mode of Co(OH)_2_.^63^ The broadness of the peak also suggests the possible overlapped contribution of the E_g_ Raman mode of CoOOH (ca. 482 cm^–1^), and the A_1g_ mode of Co_3_O_4_ and CoO (ca. 690 cm^–1^).^63,77,78^ Additionally, a second-order two-phonon Raman scattering in the 1000–1100 cm^–1^ spectral range is observed and attributed to characteristic phonon dispersion (*D*) of CoO.^78^ These observations are consistent with the redox features shown in Figure 1d pointing to the stabilization of higher oxidation states of catalytically active metals after the initial oxidation event, fostered by the presence of iron.^66^

Further insights into the chemical composition of the electrodes were obtained from X-ray photoelectron spectroscopy (XPS) analysis. It is worth noting that the XPS analysis of materials containing transition metals is often challenging due to the complexity of 2p peaks line shapes featuring peak asymmetry, multiplets splitting, and the presence of shakeup satellites and plasmon loss structures.^79,80^Figure 3d shows the Co 2p core level signal, notably with a main Co 2p_3/2_ contribution at ca. 780.7 eV, characteristic of cobalt(II) porphyrins on the reference **sCoDPP** thin film.^81^ Interestingly, the XPS Co 2p core level spectrum of the as-deposited **pCoDPP** thin film displays a broader and slightly shifted Co 2p_3/2_ region peak (Δ ≈ 0.2 eV), which can be ascribed to the presence of both Co(II) and Co(III) porphyrins, supporting the isomeric valence nature of the cobalt porphyrin radical cations formed during the oCVD process, namely, [Co(II)DPP]^+^ and [Co(III)DPP]^+^. As previously reported by our group on the investigation of the oCVD reaction of metallo-5,15 dimesityl-substituted porphyrins (*M*DMP), the porphyrin oxidative polymerization can compete with the oxidation of its central metal cation, with particular relevance for cobalt(II) and iron(III) metal centers.^60^

One should note that, in the case of the **pCoDPP** thin film in which iron-containing compounds are retained after the oCVD reaction, the photoelectron emission originating from the Fe 2p and Co 2p core levels overlap with the Auger Co LMM and Fe LMM signals, respectively, when using an Al Kα X-ray source.^80^ This compromises the detailed chemical speciation, specifically for cobalt and iron elements in the as-deposited and EC-aged samples. As depicted in Table S2 of the Supporting Information, the atomic percentage of cobalt, estimated from the Co 3s core region, remains constant in the **pCoDPP** electrode surface before and after EC-aging (ca. 2.3%) despite notable structural and catalytic performance differences. Nonetheless, in Figure 3d, one can observe a shift toward lower energies of the main Co 2p_3/2_ peak to 779.8 eV, supporting the formation of mixed Co(II)/Co(III) oxide species after EC-aging and catalytic operation shown in Figure 1d.^63,82^ These observations, along with HIM-SIMS and Raman spectroscopy analysis (Figure 3a,b), evidence the formation of new and catalytically active Co(Fe)O_*x*_ phase after EC-aging instead of the initial fused porphyrin conjugated polymer. On the other hand, the as-prepared sublimed **sCoDPP** and the oCVD **pCoDPP** thin films do not exhibit any prominent satellite peaks (Figure 3d), consistently with a molecular cobalt environment.^38^ Conversely, the EC-aged **pCoDPP** thin film before and after rinsing with acetone depicted well-defined satellites located at ca. +6 eV from the main Co 2p_3/2_ signal (denoted as (*) in Figure 3d), a characteristic feature of Co(II) in an oxygen environment, often used as a signature of CoO phases.^38,82,83^ Noteworthy, the presence of higher oxidation states cannot be discarded, as Co(III) oxides tend to exhibit only very weak satellites in the Co 2p region. At the same time, the Co 2p binding energy (specifically CoOOH) is indistinct from Co(II) in CoO.

Regarding the Fe 2p core level signals (Figure S7), the as-deposited **pCoDPP** thin film depicted a main Fe 2p_3/2_ peak located at ca. 711.5 eV, characteristic of iron(III) chloride^84^ from unreacted oxidant disseminated at the electrode surface after oCVD (Figure 3a). After EC-aging, the signal is shifted to lower binding energies, up to ca. 710.0 eV, which is characteristic of iron compounds with an oxide environment.^79,84^ The complexity of the photoemission spectra of cobalt–iron compounds discussed above, added to the presence of an intense Sn 3p signal from the FTO substrate in the Fe 2p region (Figure S7), also makes the quantification of iron challenging, in particular, for the EC-aged samples. Both the Fe 2p_1/2_ and Fe 3p core regions were used for quantification with comparative purposes, leading to similar results (Table S2). It is worth noting that from the Fe 3p core region the Fe atomic percentage is slightly overestimated due to the proximity of the Co 3p core region, with more significance in the EC-aged sample. Notably, the initial amount of Fe (ca. 2.5%, mainly related to FeCl_*x*_ clusters residual from the oCVD reaction) on the as-deposited **pCoDPP** thin film drastically drops to quantities below 1% after EC-aging. Nonetheless, a low amount of Fe, even trace, can significantly impact the catalytic activity of CoO_*x*_ species.^53,55,63,85^

On the other hand, the analysis of the XPS spectrum at the Cu 2p core region of the as-prepared **pCoDPP** thin film prepared using CuCl_2_ as the oxidant shows the Cu 2p_3/2_ peak positioned at 935.3 eV (Figure S8), the characteristic of CuCl_2_.^84^ Notably, the Cu 2p_3/2_ peak shifts toward lower binding energies, expressly to 933.1 eV, on the EC-aged **pCoDPP (CuCl**_**2**_**)** thin film suggesting the formation of copper oxide species, specifically CuO, after the electrochemical treatment.^82,84^ The formation of mixed copper–cobalt oxides cannot be ruled out per se, as CuCo_2_O_4_ and CuO have virtually identical photoemission features, preventing the differentiation of the two phases.^86^

Additionally, the XPS spectrum of the N 1s core level region also provides clear evidence of the fate of the initial **pCoDPP** polymeric structure after EC-aging. Both the as-prepared **pCoDPP** and reference **sCoDPP** thin films show the characteristic pyrrole nitrogen environments (N–Co at 399.0 eV),^38,60^ confirming the retention of the Co metal cation at the porphyrin core (Figure 3e). Besides, the signal exhibits a peak contribution at higher binding energy, pointing to the presence of amino groups (N–H), consistent with the presence of both Co(II) and Co(III) porphyrins. Notably, the N 1s signal recorded after EC-aging becomes very weak, consistent with the C–N region mapping in Figure 3b, evidencing the deterioration of the porphyrin conjugated polymer after electrochemical aging. The residuals of the organic moiety were rinsed with acetone, as confirmed by suppression of the N 1s core region signal. In contrast, the Co 2p and Fe 2p signals remain barely unaltered, pointing to the retention of both metals at the electrode surface and not further linked with the porphyrin conjugated polymer precatalyst.

### Effect of Porphyrin Substituents on the Formation of Co(Fe)O_*x*_ Species

**Figure 4:**
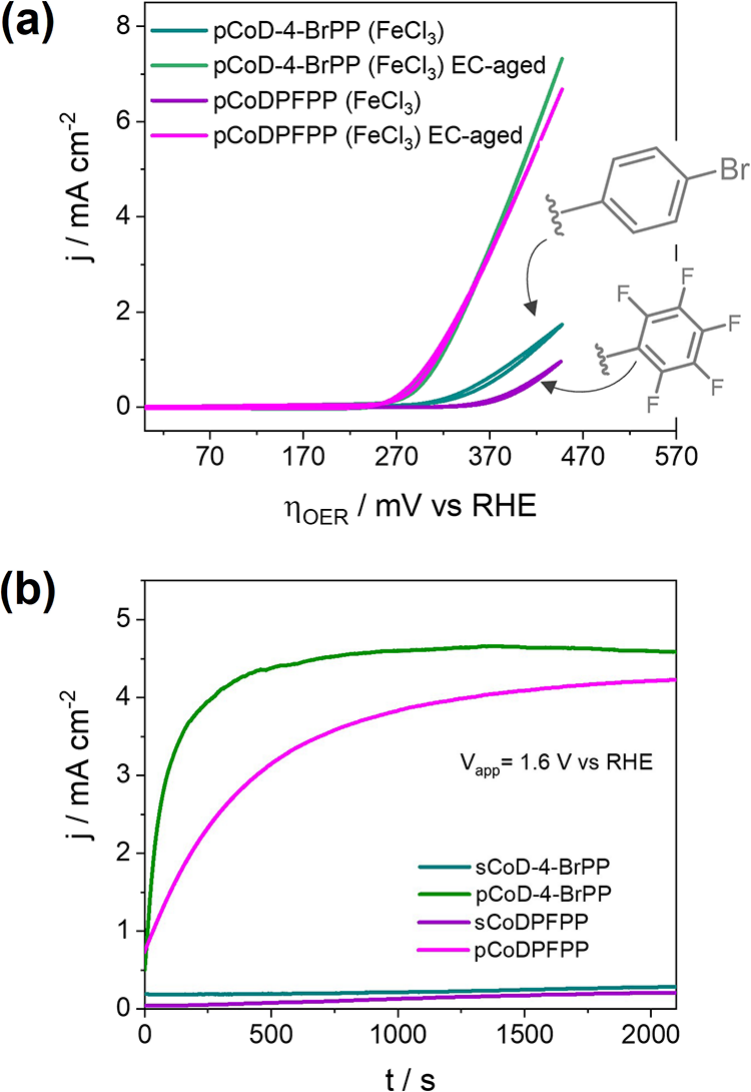
(a) CVs of **pCoD-4-BrPP** and **pCoDPFPP** before after EC-aging. (b) Chronoamperometry measurement of sublimed (monomers) and oCVD (conjugated polymers) thin films prepared from CoD-4-BrPP and CoDPFPP. A significant current density increase is observed for **pCoD-4-BrPP** and **pCoDPFPP** upon EC-aging in comparison to their **sCoD-4-BrPP** and **sCoDPFPP** references.

To further study the electrochemical transformation of cobalt porphyrin conjugated polymers into the active Co(Fe)O_*x*_ species, we investigated three different porphyrin substituents (Scheme 1). Specifically, the EC-aging treatment was applied to fused cobalt porphyrin conjugated polymers bearing either 4-bromophenyl or pentafluorophenyl substituents (**pCoD-4-BrDPP** and **pCoDPFPP**, respectively). Interestingly, the CV of the EC-aged oCVD thin films prepared from the three different cobalt porphyrins (CoDPP, CoD-4-BrDPP, and CoDPFPP) and FeCl_3_ almost overlaps with overpotentials of 260 mV (Figures 1b and 4a). This suggests that, regardless of the substituent, the fused cobalt porphyrin conjugated polymer thin films convert to active Co(Fe)O_*x*_ catalysts. This observation is confirmed by XPS analysis of **pCoD-4-BrDPP** and **pCoDPFPP** before and after EC-aging, depicting similar behaviors to **pCoDPP** in the Co 2p, Fe 2p, and N 1s regions (Figure S9). Nonetheless, the recorded chronoamperometry measurements for **pCoDPP**, **pCoD-4-BrDPP**, and **pCoDPFPP** show notable differences (Figure 4a). In particular, the time required to reach a steady current density is significantly longer for **pCoDPFPP**, indicating a different dynamic of the transformation. Such a behavior can be explained by the influence of substituent on the fused cobalt porphyrin conjugated polymer conformation synthesized by oCVD. Indeed, the blocked *ortho*-positions of the pentafluorophenyl group prevent any extension of the π-conjugated system via intramolecular cyclization, directly affecting the electric and electronic properties.

**Figure 5:**
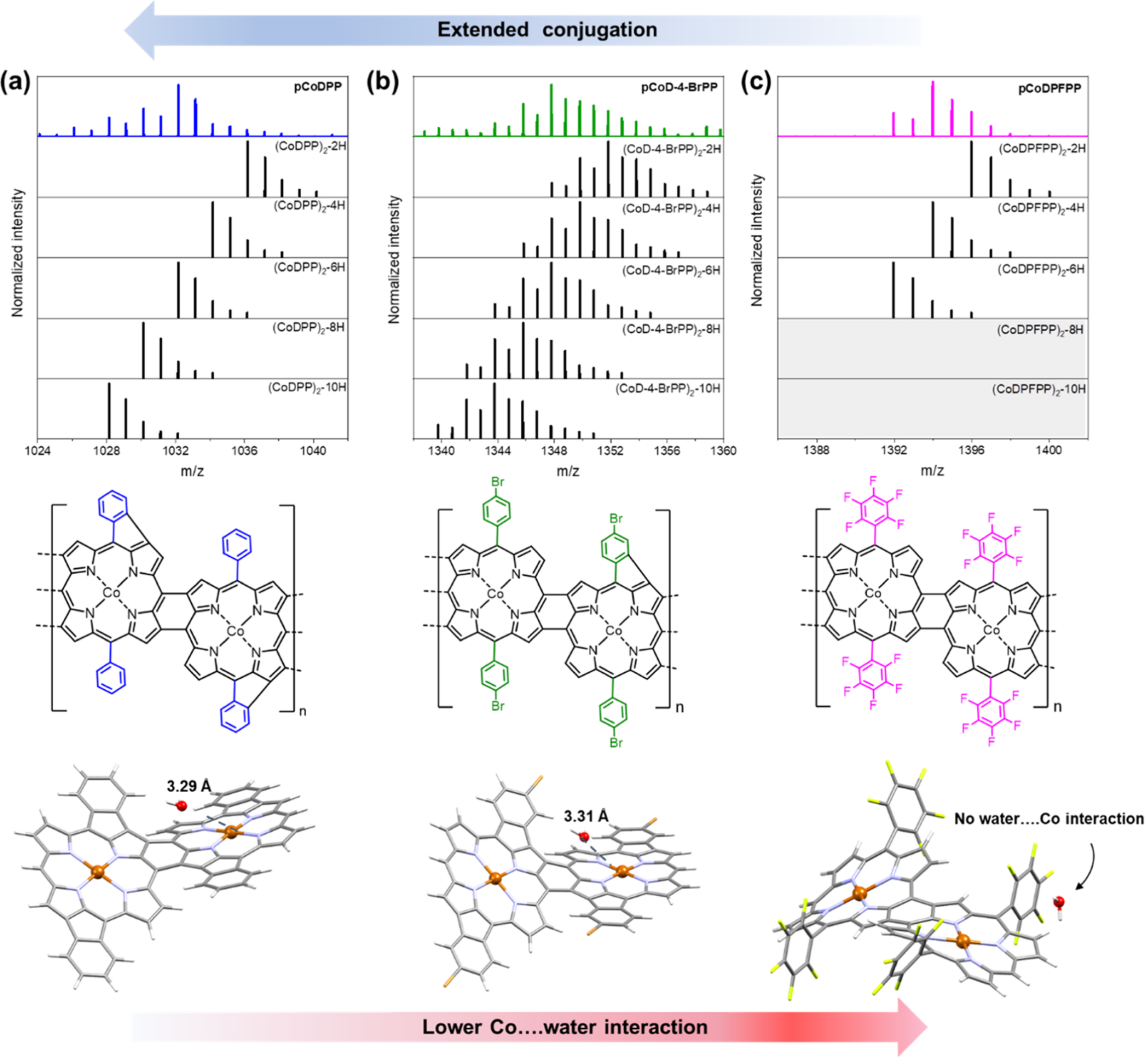
Top row: LDI-HRMS spectra of the oCVD (conjugated polymers) thin films (colored lines) in the dimer region and simulated patterns corresponding to [(CoDRP)_2_ -(H_2_)*_m_*]^+^ (black lines). (a) **pCoDPP**, (b) **pCo-4-BrDPP**, and (c) **pCoDPFPP.** Middle row: Representation of formed fused porphyrin dimers according to the extension or not of π-conjugated system due to the occurrence (**pCoDPP** and **pCo-4-BrDPP**) or absence (**pCoDPFPP**) of intramolecular C–C coupling between the *ortho-*position of the aryl substituent and β-positions of the porphyrin macrocycle. Bottom row: Water docking distance DFT calculations showing the interaction of the optimized fused porphyrin dimers with water molecule.

LDI-HRMS analysis illustrates the significant influence of substituents on the extension of the π-conjugated system of the conjugated polymer thin films (Figure S11). As shown in Figure 5a (top row), the comparison of experimental and simulated HRMS patterns of the as-prepared **pCoDPP** thin film in the dimer region evidenced the loss of up to eight and ten hydrogen atoms. The elimination of four to five hydrogen pairs for doubly and triply fused porphyrin dimers is rendered possible by the occurrence of one to several intramolecular C–C couplings between the phenyl groups and the porphyrin macrocycle of CoDPP. A similar behavior is also expected for the 4-bromophenyl-substituted porphyrin, which possesses free *ortho*-positions on its aryl group. However, a lower extent of intramolecular oxidative dehydrogenative coupling is observed for **pCoD-4-BrPP** (Figure 5b), potentially due to the electron-withdrawing character of bromide. In contrast, the complete substitution of the aryl group in **pCoDPFPP** fully impedes the formation of any additional intramolecular C–C bonding, preventing further extension of the π-conjugated system (Figure 5c).

The UV–Vis-NIR spectra of the as-prepared **pCoDPP** and **pCoD-4-BrPP** thin films (Figure S10) show broadened Soret and Q-bands as well as significantly more pronounced absorption in the NIR region. This enhanced absorption in the NIR region indicates the formation of multiply-fused porphyrin tapes, in agreement with previous studies from our group.^57,59,60,62^ Former DFT calculations pointed to mixed a_1u_/a_2u_ molecular symmetry of the radical cation promoting the formation of double β-meso/meso−β and triple β–β/*meso–meso*/β–β linkages between the cobalt porphyrin units.^60^ Furthermore, the UV–vis-NIR absorption spectra of **pCoDPP** and **pCoD-4-BrPP** remain unaltered after rinsing with dichloromethane, confirming the formation of highly conjugated and longer oligomers (Figure S10). In contrast, the UV–vis-NIR absorbance spectrum obtained for the as-prepared **pCoDPFPP** thin film show fewer discrepancies with its respective reference sublimed **sCoDPFPP** thin film. Precisely, no significant shift of the Soret and Q-bands and a rather weak absorption in the NIR region were observed (Figure S10), suggesting a lower efficiency of the oxidative dehydrogenative coupling reaction for CoDPFPP.

Previous studies evidenced the role of intermolecular and intramolecular oxidative dehydrogenative coupling on the extension of the π-conjugated systems and the induced planarity of the fused porphyrin tapes, with benefits on the electric and optoelectronic properties and, therefore, on the photo- and electrocatalytic performances of fused porphyrin conjugated polymers.^61^ Expansion of the π-conjugated system is known to raise the energy of the HOMO level and lead to a low excitation gap in directly fused porphyrins tapes.^27^ Consequently, the intermolecular and intramolecular oxidative dehydrogenative coupling of metalloporphyrins by oCVD yields a broadening and shifting of the valence band edge toward lower binding energies compared to their respective sublimed reference thin films.^62^ Consistently with the UV–vis-NIR analysis, **pCoDPP** shows the most significant reduction of HOMO, with a decrease from 1.20 to 0.74 eV upon polymerization, estimated from the analysis at the valence band region of the XPS spectra (Figure S12). The enhanced conjugation in **pCoDPP** is responsible for a higher electron delocalization that results in superior conductivity (4.65 × 10^–4^ S cm^–2^) in comparison to **pCoD-4-BrPP** (1.16 × 10^–6^ S cm^–2^), while **pCoDPFPP** shows the lowest value (4.29 × 10^–7^ S cm^–2^) (Figure S13). Based on these observations, a higher electrocatalytic activity might be anticipated from **pCoDPP** and a lowest from **pCoDPFPP**, in agreement with the initial performance depicted in Figures 1b and 4a. Overall, these properties are also expected to impact the formation of cobalt oxide species by promoting a more significant charge transfer toward the oxidation of the cobalt centers.

Our recent study on nickel(II) porphyrin conjugated polymers bearing various phenyl substituents evidenced the crucial influence of the molecular conformation of fused metalloporphyrin conjugated polymers on the interaction between the reactant and the catalytic site.^25^ Therefore, DFT calculations were performed to visualize the structure and frontier molecular orbitals of representative doubly and triply linked dimers (Figure S14). The optimized structures show an increase structural planarity with increase of intermolecular and intramolecular oxidative dehydrogenative coupling, in accordance with our latest report on fused nickel(II) porphyrin conjugated polymers.^25^ For example, triply linked dimers are comparatively more planar than structurally twisted doubly linked dimers. Moreover, intramolecular cyclization in (CoDPP)_2_ and (CoD-4-BrPP)_2_ dimers induces the phenyl and bromophenyl substituents to align in plane respective to the cobalt porphyrin core, whereas, in the case of (CoDPFPP)_2_, the pentafluorophenyl group arranges nearly perpendicularly to the cobalt porphyrin core owing to the absence of intramolecular cyclization. These conformational differences imply different electronic environment around the active cobalt center, which can potentially influence its interaction with water and further dynamics for the formation of metal oxo intermediaries.

As shown in Figure 5 (bottom row), the docking studies indicate appropriately positioned water molecules near the cobalt center of the optimized structures of CoDPP and CoD-4-BrPP dimers. The minimum Co···O water distance is 3.29 Å in doubly fused (CoDPP)_2_ and 3.31 Å in doubly fused (CoD-4-BrPP)_2_, considering intramolecular cyclization (water docking distance for the respective monomer and doubly fused dimers are provided in the Supporting Information, Figure S15). However, this value increases to 3.65 Å when no intramolecular cyclization is considered (Figure S16). In contrast, the water molecule could be seen nowhere close to the metal center of doubly fused (CoDPFPP)_2_, supporting our findings on the lower activity of **pCoPFPPP**. Overall, the discrepancies in the dynamics of conversion of **pCoDDP**, **pCoD-4-BrDPP**, and **pCoDPFPP** highlight the significance of an extended π-conjugated system for the conversion of cobalt porphyrins into catalytically active cobalt oxides species.

A prospective advantage of this study is the straightforward integration of the fused porphyrin conjugated polymer precatalyst via oCVD, followed by their in situ electrochemical conversion to highly transparent catalytically active species, onto photoabsorbing materials for the preparation of photoelectrochemical cell combining high catalytic performances to low photon absorption losses.^87^ Furthermore, the present findings along with our recent report on the preparation of heterometallic porphyrin conjugated polymers,^88^ paves the way to the development and engineering of bi- and multimetallic polymer-derived catalysts for the selective catalytic synthesis of fine chemicals.

## Conclusions

In this work, a highly transparent and active Co(Fe)O_*x*_ catalyst for OER was prepared from the in situ electrochemical transformation of cobalt porphyrin conjugated polymers coated on FTO substrates by oCVD, using FeCl_3_ as oxidant. The decreased Tafel slope and disappearance of precatalytic cobalt oxidation peaks in the CVs, pointing to the prevalence of higher oxidation states for the electrodes derived from the conjugated polymers, evidenced the synergic function of both polymerization and presence of iron(III) used in oCVD in the formation of catalytically active phases. These results confirmed the instability of cobalt porphyrins, including fused cobalt porphyrin conjugated polymers, under alkaline OER conditions. Irrespective of the porphyrin polymerization or porphyrin substituents, electrochemical treatment under the OER conditions yields the formation of ultrathin and transparent cobalt oxide species acting as the true catalyst.

## Supporting Information Available

Experimental details on samples preparation and characterization and theoretical methods; electrochemical and structural characterizations; and optimized simulated structures and parameters of DFT calculations (PDF)
