# Supplementary material for: A Polymer-Derived Co(Fe)Ox Oxygen Evolution Catalyst Benefiting from the Oxidative Dehydrogenative Coupling of Cobalt Porphyrins
Source: ACS Catal. 2023 Nov 8;13(22):15182–93. doi: 10.1021/acscatal.3c02940 (PMC10660665; doi:10.1021/acscatal.3c02940)
Supplement: Supplementary file 1 — cs3c02940_si_001.pdf [file cs3c02940_si_001.pdf]

Supporting Information

A Polymer-Derived Co(Fe)O_x Oxygen Evolution Catalyst Benefiting from the Oxidative Dehydrogenative Coupling of Cobalt Porphyrins.

Drialys Cardenas-Morcoso†, Deepak Bansal†, Max Heiderscheid†, Jean-Nicolas Audinot†, Jérôme Guillot†, Nicolas D. Boscher†**

[†]Materials Research and Technology Department, Luxembourg Institute of Science and Technology, 28 Avenue des Hautes-Fourneaux, Esch-Sur-Alzette, Luxembourg

*drialys.cardenas@list.lu, nicolas.boscher@list.lu

Contents

Experimental and theoretical methods 4

Scheme S1. Schematic of the oCVD reactor used for the preparation of the porphyrin-based thin films from the gas-phase. 7

Table S1. Deposition conditions for the chemical vapor deposition of the porphyrin thin films. 7

Figure S1. LDI-HRMS spectra of the reference **sCoDPP** (sublimed) and oCVD **pCoDPP** coatings from CoDPP. 8

Figure S2. CV of the reference **sCoDPP** electrode before (black) and after EC-aging (red)... 9

Figure S3. CV of the **pCoDPP** electrode before (black) and after EC-aging (red) and EC-aged **pCoDPP** electrode after acetone rinsing (blue). 9

Figure S4. (a) UV-VIS-NIR absorption spectra and **(b)** LDI-HRMS spectrum of **pCoDPP ($CuCl_2$)** thin film, showing the formation of fused cobalt porphyrin oligomers from the oCVD reaction of CoDPP and $CuCl_2$ as the oxidant agent. 10

Figure S5. (a) LSV of the as-prepared and EC-aged **pCoDPP** thin films prepared using $FeCl_3$ and $CuCl_2$ as oxidant agent during oCVD. **(b)** Chronoamperometry performed at 1.6 V vs RHE (EC-aging) on the **pCoDPP** thin film prepared using $CuCl_2$. 11

Figure S6. (a) Chronoamperometry recorded during the gas evolving measurements on the EC-aged **pCoDPP** electrode, performed at 1.6 V vs RHE applied potential in 1M KOH. **(b)** Calculated oxygen evolution rate and faradaic efficiency. 12

Figure S7. XPS spectra on the Fe 2p core region of the as-prepared **pCoDPP** (using $FeCl_3$ as the oxidant), after EC-ageing and rinsed with acetone. 13

Figure S8. XPS spectra on the Cu 2p core region of the as-prepared **pCoDPP** (using $CuCl_2$ as the oxidant) and after EC-ageing. 13

Table S2. Elemental composition from XPS analysis of fresh and EC-aged coatings. 14

Figure S9. XPS spectra on the **a)** Co 2p, **b)** Fe 2p and **c)** N 1s regions of the **pCo-4-BrDPP** and **pCoDPFPP** thin films as prepared and after EC-aging. 15

Figure S10. (a), (c) and **(e)** Digital pictures of the sublimed reference (left side) and oCVD (right side) coatings as deposited, and after rinsing a section of the films with dichloromethane (DCM). The DCM-rinsed area is marked inside a dash-lined box. **(b), (d)** and **(f)** Absorbance spectra of the sublimed porphyrin monomers (s), and the oCVD polymer films (p) as deposited and after rinsing with DCM. 16

Figure S11. LDI-HRMS spectra of the reference (sublimed) and oCVD coatings from **(a)** CoD-4-BrPP and **(b)** CoDPFPP. 17

Figure S12. XPS spectra on the valence band region of the reference (sublimed) (black dots) and oCVD (red dots) coatings. 18

Figure S13. Lateral conductivity of the oCVD thin films coated on OFET substrates, calculated from the 2-points probe method (length of the chip= 2.5 µm) 18

Figure S14. Optimized structures of doubly and triply fused cobalt porphyrin dimers, showing the distribution of frontier molecular orbitals. 19

Figure S15. Water docking distance calculations showing the interaction of water molecule with the optimized **a-c)** monomers and **d-f)** triply fused dimers, considering intramolecular cyclization. 20

Figure S16. Water docking distance calculations showing the interaction of water molecule with the optimized **a-b)** monomers, **c-d)** doubly and **e-f)** triply fused dimers, without considering intramolecular cyclization. 20

Table S3. Coordinates for doubly linked CoDPP dimer. 21

Table S4. Coordinates for triply linked CoDPP dimer. 24

Table S5. Coordinates for doubly linked CoD-4-BrPP dimer. 28

Table S6. Coordinates for triply linked CoD-4-BrPP dimer. 31

Table S7. Coordinates for doubly linked CoDPFPP dimer. 35

Table S8. Coordinates for triply linked CoDPFPP dimer. 39

References 43

Experimental and theoretical methods

Oxidative Chemical Vapor Deposition (oCVD)

The oCVD reaction was performed in a custom-built oCVD reactor, represented in **Scheme S1**, and described elsewhere[1-4]. *5,15* di-substituted cobalt(II) porphyrins with different substituents (**Scheme 1** in the main manuscript), were obtained from PorphyChem (98%) and used without further purification. Based on previous reports,[1, 3] iron(III) chloride (97%, Sigma-Aldrich) was chosen as the oxidant. **Table S1** summarizes the deposition conditions used for each porphyrin investigated. The temperature used to sublime the oxidant was 170 °C in all cases. Alternatively, copper(II) chloride (anhydrous, powder, ≥99.995% trace metals basis, Sigma-Aldrich) was used as oxidant for control experiments, with a sublimation temperature of 300 °C.

Glass microscope slides (Menzel-Gläser Superfrost®), interdigitated chips (OFET Gen4, Fraunhofer) and fluoride-doped tin oxide coated glass (FTO TEC 15, Ossila) were used as substrates. Prior deposition, all the substrates were cleaned with absolute ethanol (99.98%, VWR chemicals®) and dried with nitrogen gas. The substrate holder was kept at 150°C for all the depositions. The pressure inside the oCVD reactor was kept at 10^{-3} mbar, under Argon (99.999 %, Air Liquide) atmosphere. The deposition time was set to 30 minutes for all experiments. Additionally, reference sublimed porphyrin monomers thin films were obtained under the same conditions, without supplying any oxidant. All the oCVD samples (as-prepared and after electrochemical characterizations) were stored under vacuum conditions until their subsequent analysis.

Thin films characterization

The ultraviolet-visible-near infrared (UV-Vis-NIR) spectra of the sublimed and oCVD films deposited on glass slides were recorded with in a Perkin-Elmer Lambda 1050 spectrometer, in the transmission (T) mode, in the 300-2500 nm wavelength interval. The absorbance (A) was calculated as: $A= -log(T)$. The as-deposited thin films were further rinsed with dichloromethane (anhydrous, ≥99.8%, Sigma-Aldrich) and the solvent-soluble fraction was measured in a transparent quartz cuvettes of 3.5 mL capacity and 1 cm of light path. The rinsed glass slides were also analyzed for comparison with the as-deposited films.

Electron Microscopy and SIMS imaging were performed with Helium-Ion Microscopy (Nanofab, Zeiss, Peabody) coupled to Secondary Ion Mass Spectrometer developed at LIST (HIM-SIMS).[5] Measurements were performed using an 25 keV He^+ beam (0.5 pA) and 25 keV Ne^+ beam (8 pA) for secondary electron (SE) and SIMS analysis respectively. The secondary ions were detected, simultaneous for each polarity, in the multicollection system. On the same region of interest, the positive ions were ^{39}K, ^{56}Fe, ^{59}Co and ^{120}Sn. In negative mode the ions recorded were ^{16}O, ^{12}C, ^{35}Cl and the cluster $^{12}C^{14}N$. EM images were acquired in a matrix of 1024x1024 pixels and a field of view of 10x10μm², with a counting time of 10μs/pixel repeated 4 time per line (line average). SIMS images were acquired at a size of 10 x 10 μm² in a matrix of 512x512 pixels (20 nm/pixel) and a counting time of 2 ms/ pixel (~8 min of acquisition per polarity).

X-ray photoelectron spectroscopy (XPS) measurements were performed a Nexsa – G2 instrument (ThermoFisher Scientific, UK) using a monochromatic Al K_α X-ray source (E =

1486.6 eV) and a 400 μm spot size. The binding energy of the spectra was referenced by fixing the carbon (C 1s) to 285.0 eV.

Raman spectra was recorded at room temperature with an inVia Raman Microscope (RENISHAW), using a 633 nm laser excitation.

Laser desorption/ionization high-resolution mass spectrometry (LDI-HRMS) measurements were performed using an AP-MALDI UHR ion source (MassTech, Inc.) coupled to an LTQ/Orbitrap Elite (ThermoScientific). In-source fragmentation (E = 70 V) was used to prevent the formation of clusters. The measurements were performed on Si wafer substrates coated either with the sublimed porphyrin monomer and oCVD film, which were directly placed on the sample holder. LDI-HRMS measurements and analysis of metalloporphyrin coatings has been described in detail elsewhere. [2-4]

The lateral conductivity of the thin films was assessed by 2-point current-voltage scans, using a microprobe station (Cascade Microtech, PM8) and a Keithley (2401) source-meter. The data were recorded by sweeping the voltage from –4 V to 4 V and back (hysteresis scan) at a scan rate of 500 mV s^{-1}, and the geometry of the channel was 2.5 μm (length, l), 10 mm (width, w) and 40 nm (height, h). The lateral conductivity was evaluated from a linear fit and the channel geometry as: $\sigma[S\ cm^{-1}] = ({}^{1}/_{R}) \times ({}^{l}/_{A})$; where ${}^{1}/_{R}$ is the slope of the current-voltage scan (Ohm's law), and A is the cross-sectional area $A\ [cm^2] = w \times h$.

Electrochemical characterizations

All the electrochemical measurements were performed using an AUTOLAB potentiostat/galvanostat, in a three-electrode cell consisting in an Ag/AgCl (3 M KCl) reference electrode, a Pt wire as a counter electrode and the fused-metalloporphyrin coating on FTO substrate as working electrode. A 1 M potassium hydroxide (ACS reagent, ≥85%, pellets, Sigma-Aldrich) solution of pH 13.6 was used as electrolyte. Cyclic voltammetry (CV) measurements were recorded at a scan rate of 50 mV s^{-1} with 5 mV step, and linear sweep voltammetry (LSV) measurements were performed at 10 mV s^{-1} scan rate. All the potentials were referred to the Reversible Hydrogen Electrode (RHE) through the Nernst equation: $V_{RHE} = V_{Ag/AgCl} + V^0_{Ag/AgCl} + 0.059\ \times pH$.

Gas evolution measurements were performed in a sealed cell coupled to an Agilent micro-gas chromatograph (GC). Prior to GC measurements, the electrolyte (1M KOH) was purged with argon, and a constant inlet argon flow of 12 mL min^{-1} was kept in the sealed cell during the experiments. The oxygen evolution was monitored every 5 min during a chronoamperometric measurement performed at 1.6 V vs RHE applied potential. The faradaic efficiency (FE) for gas evolution at the electrode surface was estimated from the ratio between the experimental and the theoretical evolved gas amount calculated with the Faraday´s Law: $n_{O_2}\ (mol) = \frac{I \cdot t}{z \cdot F}$, where I is the recorded current by chronoamperometry, t the time, z is the number of transferred electrons, and F is the Faraday constant (96 485 C mol^{-1}).

Density functional theory calculations.

All the structures were optimized using Orca version 5.0.1.[6-8] The DFT calculations were performed using the BP86[9, 10] functional with Karlsruhe valence triple-zeta basis set "def2-

TZVP"[11-13] and Weigend's auxiliary basis set[14]. Dispersion effects were considered by Grimme approximation 'D3'.[15, 16] To simply speed up the iteration, RIJCOSX approximation is included.[17, 18] The optimized geometries were confirmed attaining local minima by confirming absence of negative frequencies after numerical frequency analysis. Docking experiments were performed using Hex 8.0 docking software.

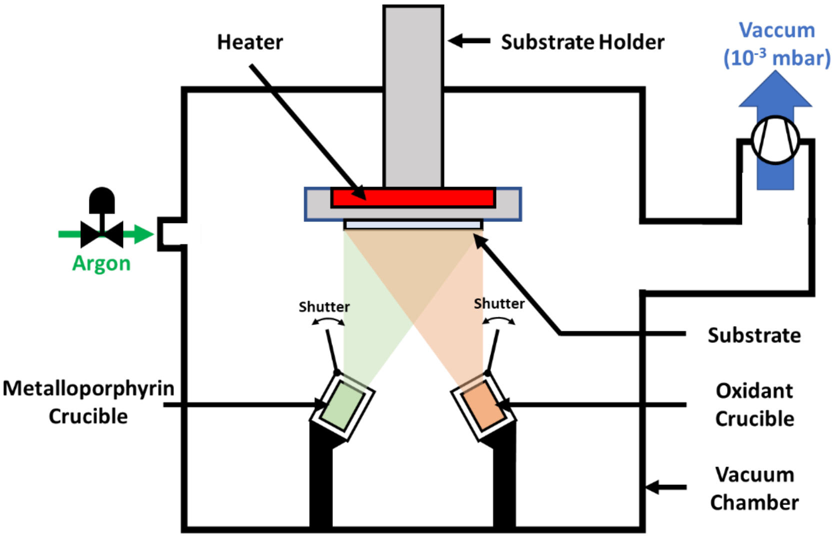

Scheme S1. Schematic of the oCVD reactor used for the preparation of the porphyrin-based thin films from the gas-phase.

Table S1. Deposition conditions for the chemical vapor deposition of the porphyrin thin films.

Thin film	T/°C (porphyrin crucible)	Amount of porphyrin consumed /mg	T/°C (oxidant crucible)	Amount of oxidant consumed /mg
sCoDPP		7.8	-	-
**pCoDPP**	250	7.6	170	112.7
**pCoDPP ($CuCl_2$)**	250	5.4	300	51.1
sCoD-4-BrPP	300	6.2	-	-
**pCoD-4-BrPP**		3.4	170	112.2
sCoDPFPP	275	13.8	-	-
**pCoDPFPP**		13.7	170	110.2

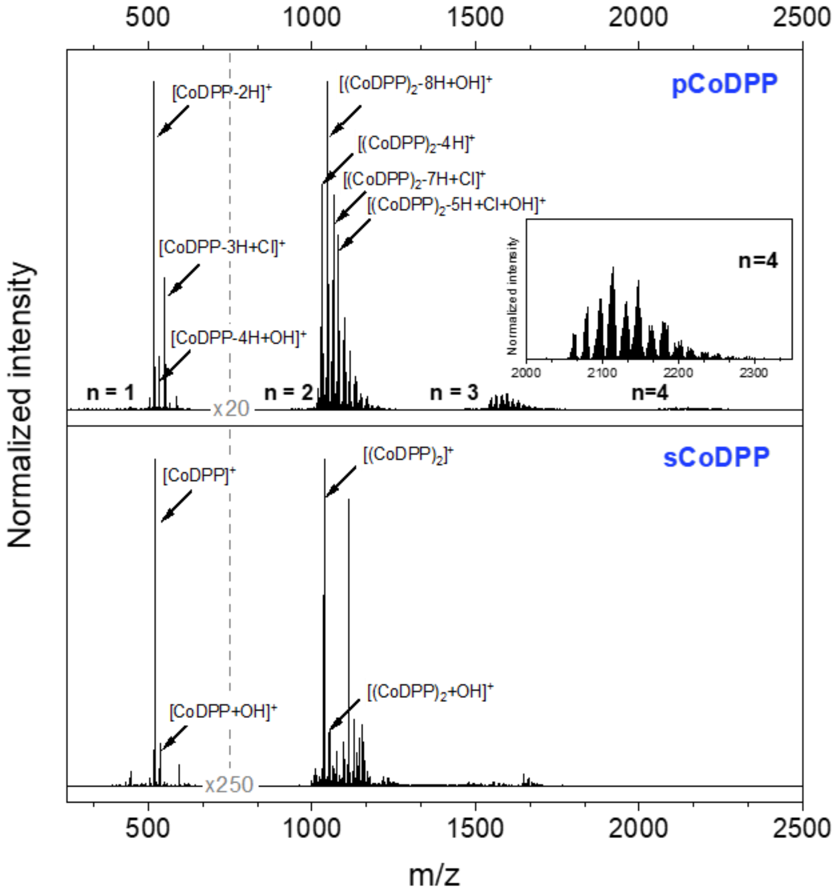

Figure S1. LDI-HRMS spectra of the reference **sCoDPP** (sublimed) and oCVD **pCoDPP** coatings from CoDPP.

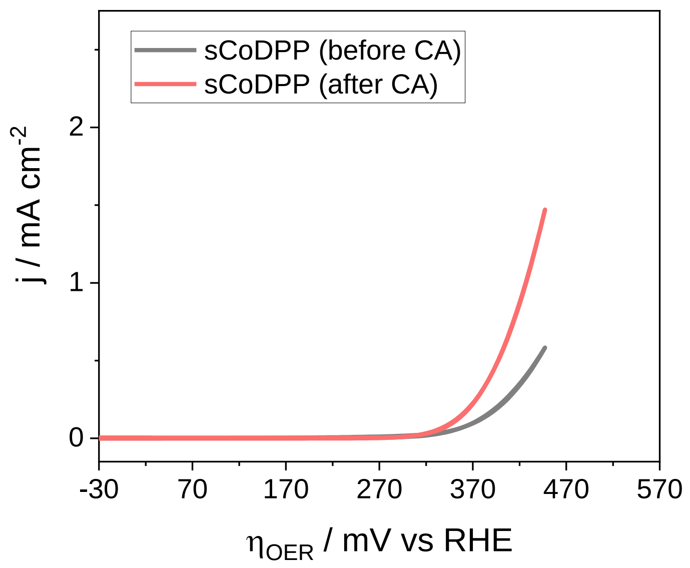

Figure S2. CV of the reference **sCoDPP** electrode before (black) and after EC-aging (red).

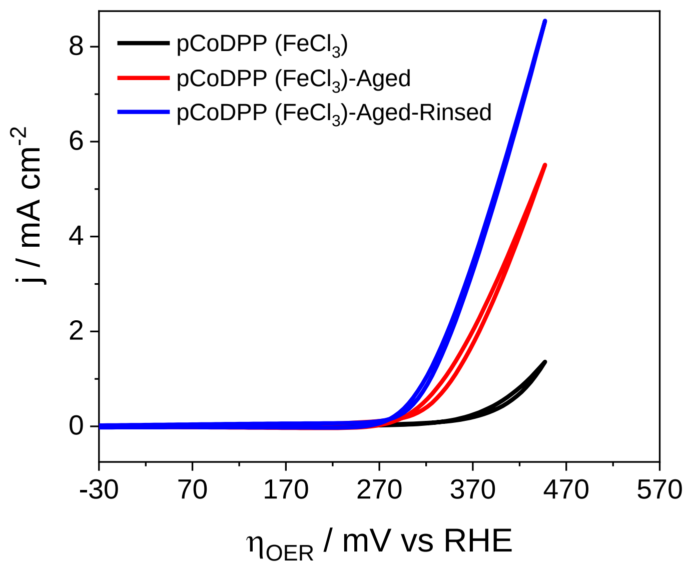

Figure S3. CV of the **pCoDPP** electrode before (black) and after EC-aging (red) and EC-aged **pCoDPP** electrode after acetone rinsing (blue).

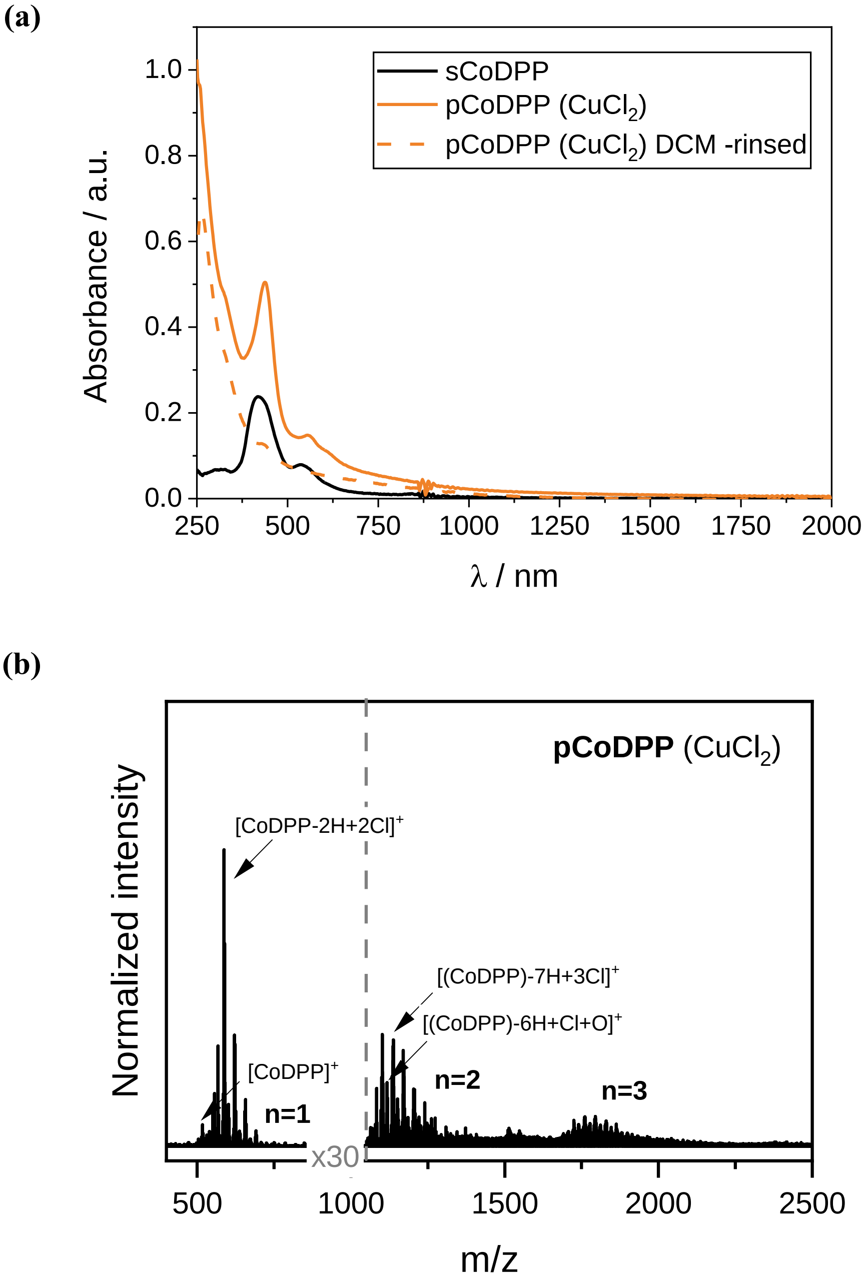

Figure S4. (a) UV-VIS-NIR absorption spectra and **(b)** LDI-HRMS spectrum of **pCoDPP ($CuCl_2$)** thin film, showing the formation of fused cobalt porphyrin oligomers from the oCVD reaction of CoDPP and $CuCl_2$ as the oxidant agent.

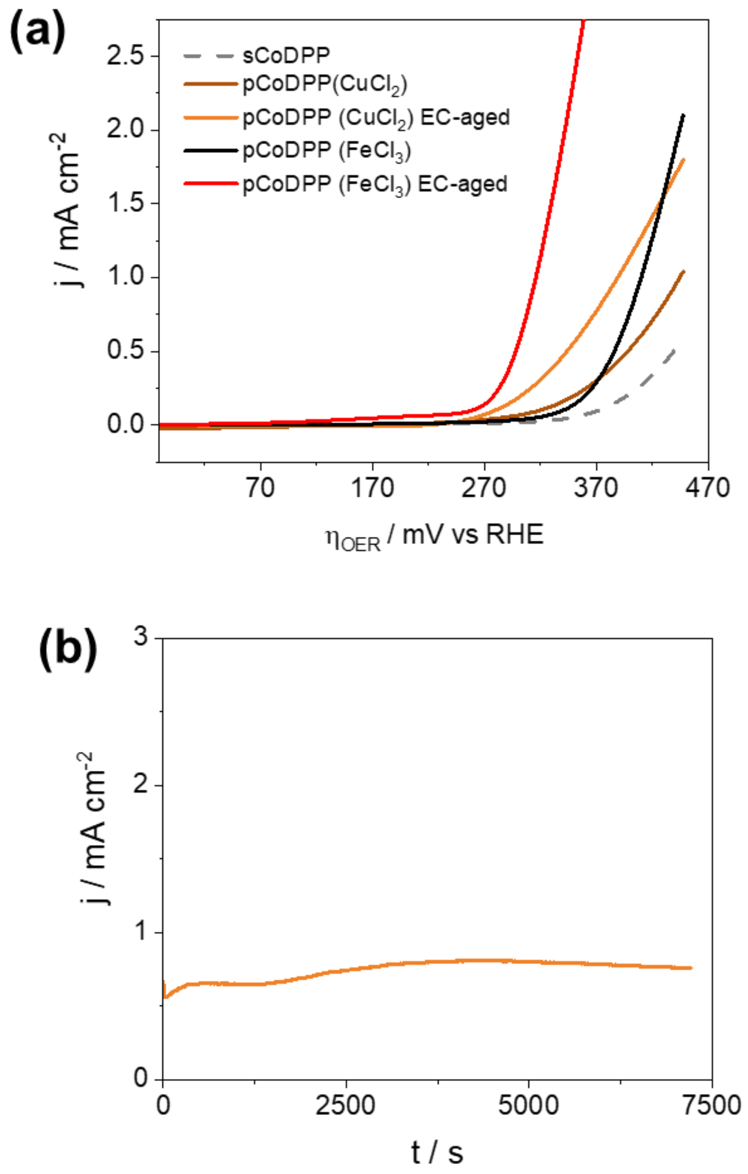

Figure S5. (a) LSV of the as-prepared and EC-aged **pCoDPP** thin films prepared using $FeCl_3$ and $CuCl_2$ as oxidant agent during oCVD. **(b)** Chronoamperometry performed at 1.6 V vs RHE (EC-aging) on the **pCoDPP** thin film prepared using $CuCl_2$.

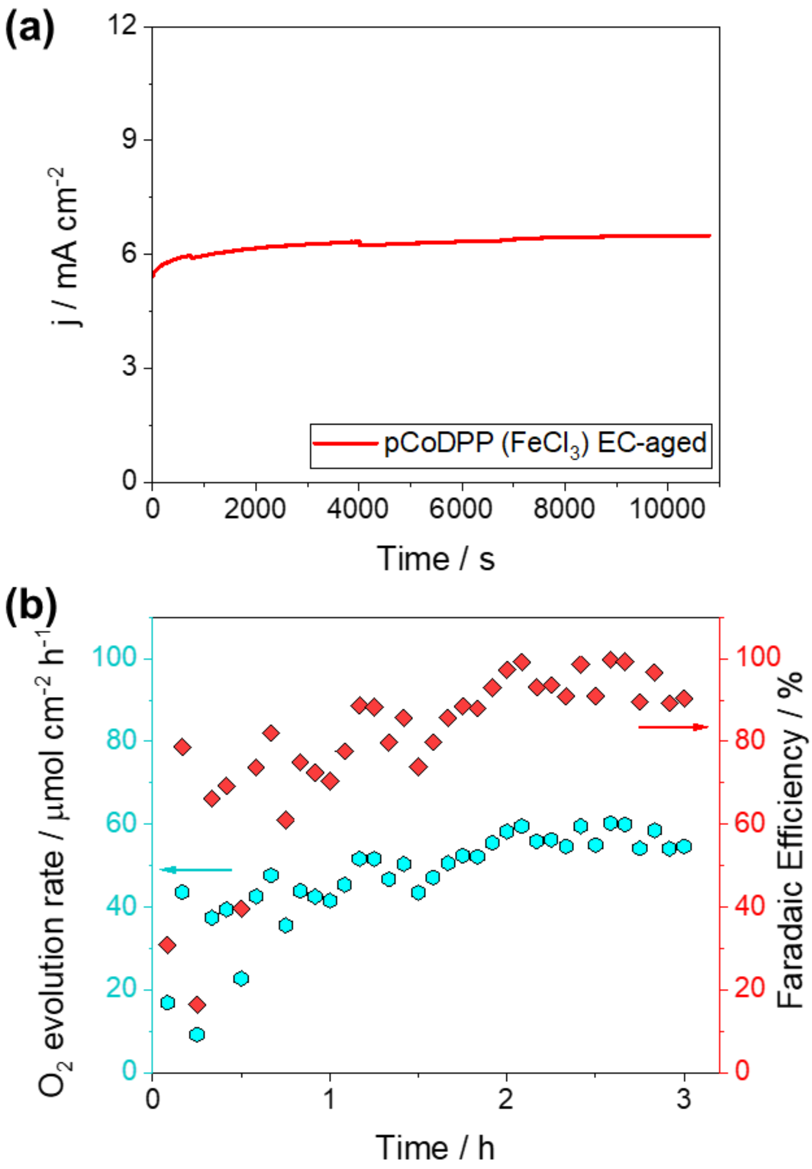

Figure S6. (a) Chronoamperometry recorded during the gas evolving measurements on the EC-aged **pCoDPP** electrode, performed at 1.6 V vs RHE applied potential in 1M KOH. **(b)** Calculated oxygen evolution rate and faradaic efficiency.

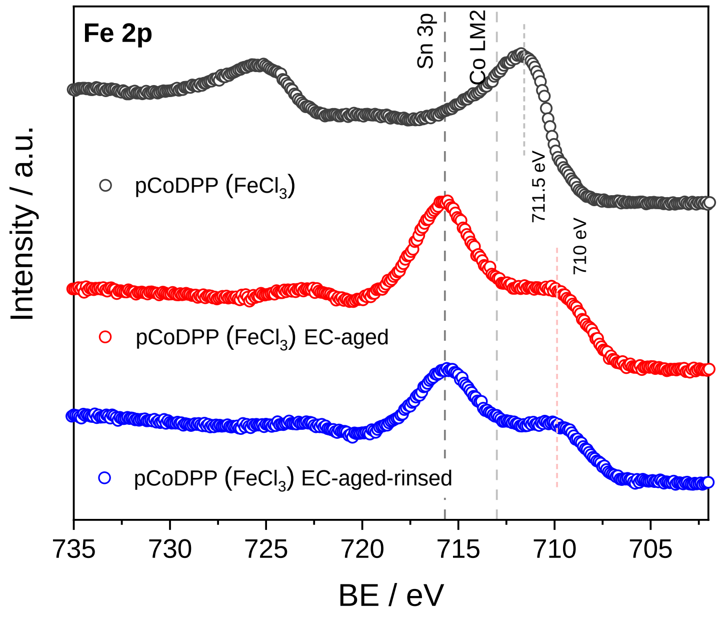

Figure S7. XPS spectra on the Fe 2p core region of the as-prepared **pCoDPP** (using $FeCl_3$ as the oxidant), after EC-ageing and rinsed with acetone.

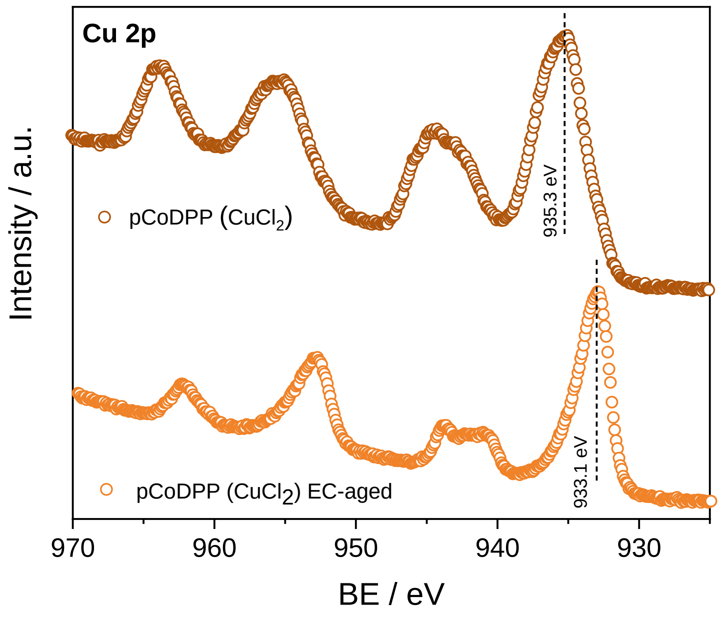

Figure S8. XPS spectra on the Cu 2p core region of the as-prepared **pCoDPP** (using $CuCl_2$ as the oxidant) and after EC-ageing.

Table S2. Elemental composition from XPS analysis of fresh and EC-aged coatings.

Sample	Co 3s %	N 1s %	C 1s %	Fe(*) %	Cu 2p %	Cl 2p %	O 1s %	Sn 3d %
**sCoDPP**	**1.7**	7.2	71.6	-	-	-	17.0	2.5
**sCoDPP EC-aged**	**1.9**	5.5	70.4	**-**	**-**	-	19.4	2.8
**pCoDPP($FeCl_3$)**	**2.3**	5.7	68.9	**2.5**†	**-**	3.3	17.3	0.0
**pCoDPP($FeCl_3$)**	**2.3**	5.6	68.7	**2.9**‡	**-**	3.3	17.3	0.0
**pCoDPP($FeCl_3$) EC-aged**	**2.4**	2.7	42.3	**<0.5**†	**-**	0.5	48.9	4.2
**pCoDPP($FeCl_3$) EC-aged**	**2.3**	2.8	41.4	**0.9**‡	**-**	0.5	48.1	4.0
**pCoDPP($FeCl_3$) EC-aged (rinsed)**	**2.5**	0.9	60.5	**0.7**‡	-	0.3	32.2	2.9
**pCoDPP($CuCl_2$)**	**2.0**	3.9	48.9	**-**	**12.8**	13.3	20.4	0.1
**pCoDPP($CuCl_2$) EC-aged**	**2.5**	0.6	50.3	**-**	**5.4**	0.4	32.9	7.8

(*) Due to overlapping between Fe $2p_{3/2}$ core region and Co LM2, Fe % is estimated from the Fe $2p_{1/2}$ (†) and Fe 3p (‡), for comparative purposes.

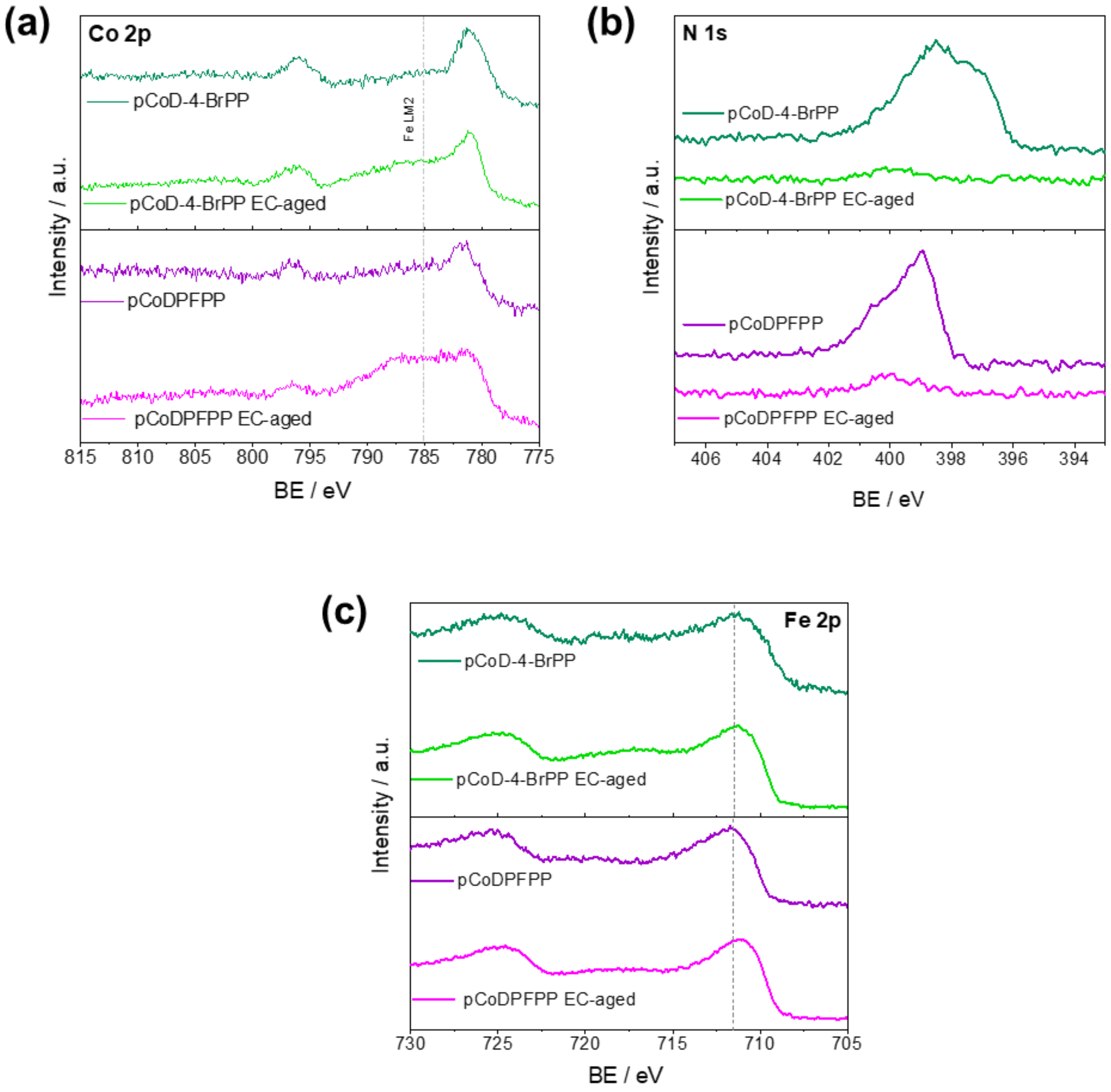

Figure S9. XPS spectra on the **a)** Co 2p, **b)** Fe 2p and **c)** N 1s regions of the **pCo-4-BrDPP** and **pCoDPFPP** thin films as prepared and after EC-aging.

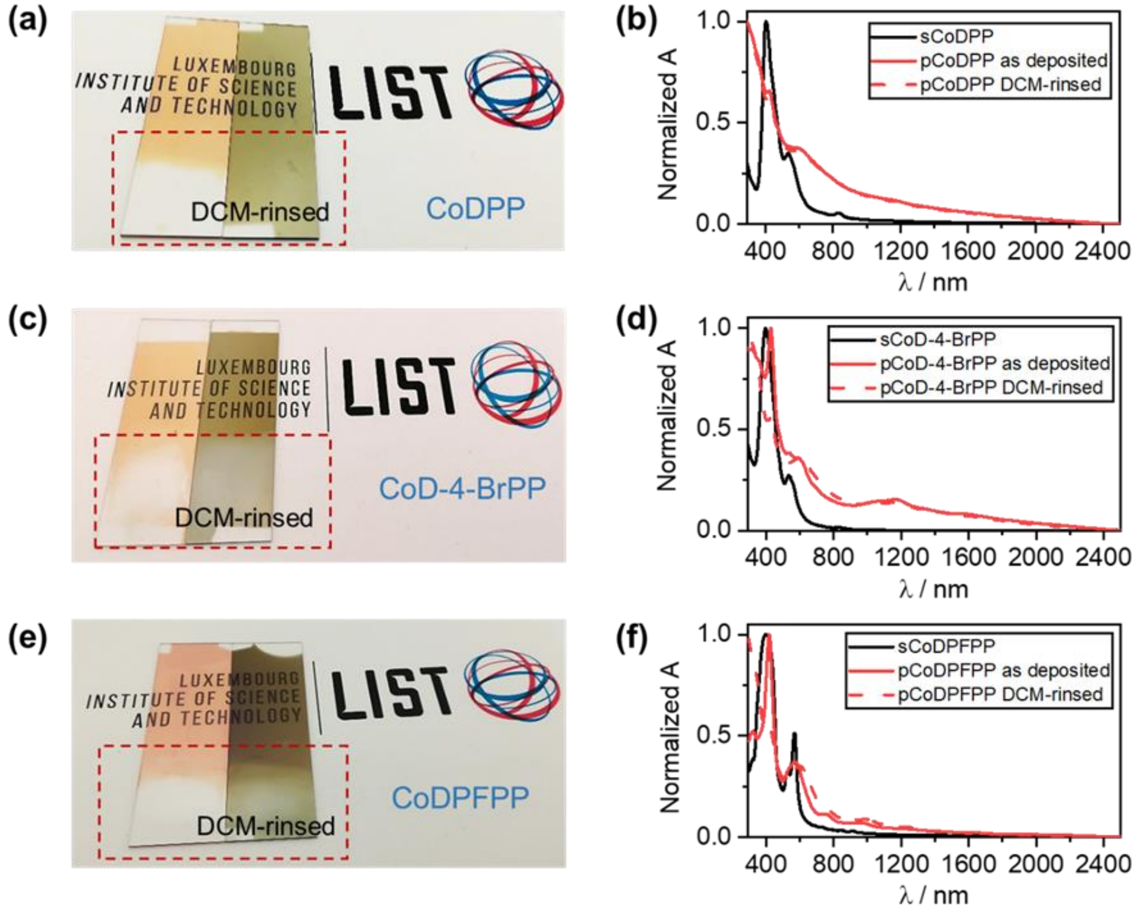

Figure S10. (a), (c) and **(e)** Digital pictures of the sublimed reference (left side) and oCVD (right side) coatings as deposited, and after rinsing a section of the films with dichloromethane (DCM). The DCM-rinsed area is marked inside a dash-lined box. **(b), (d)** and **(f)** Absorbance spectra of the sublimed porphyrin monomers (s), and the oCVD polymer films (p) as deposited and after rinsing with DCM.

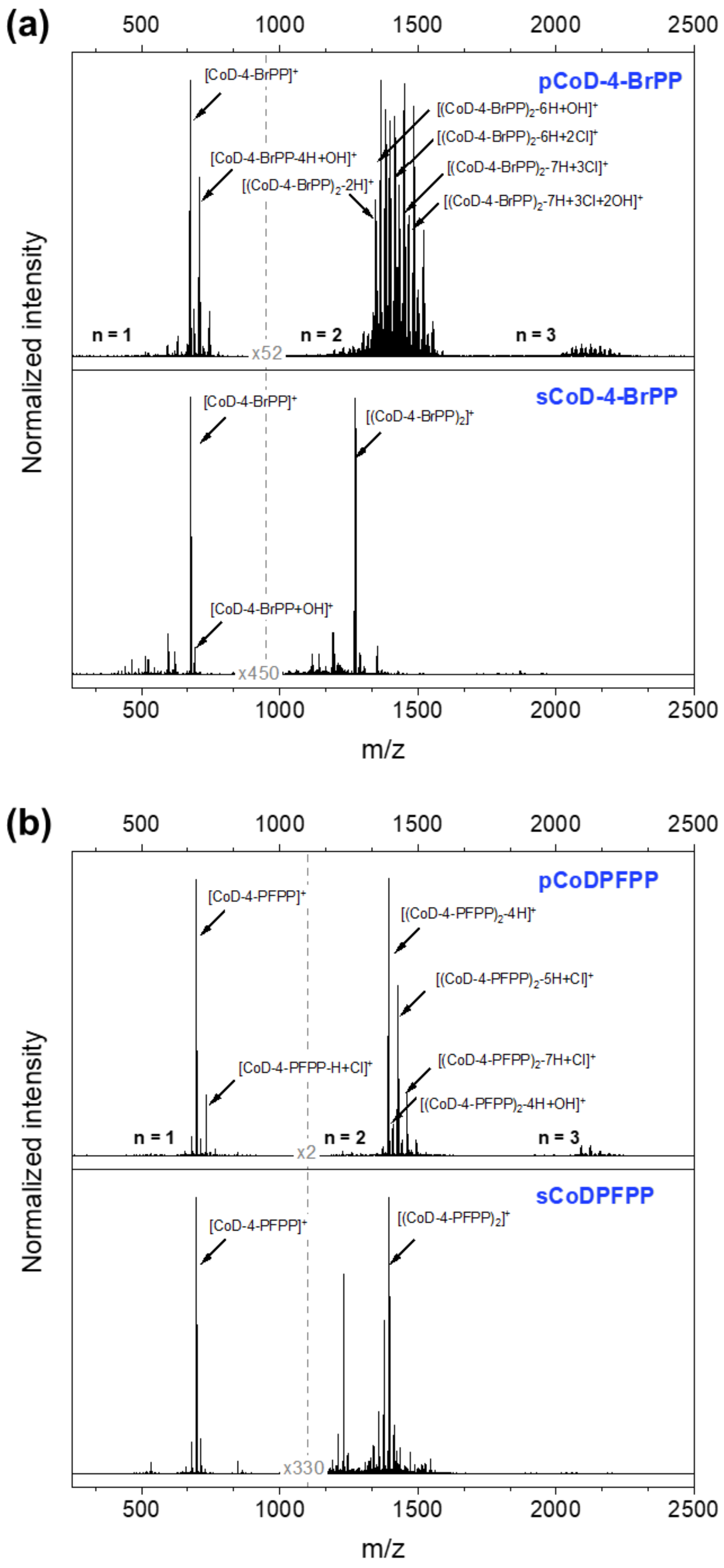

Figure S11. LDI-HRMS spectra of the reference (sublimed) and oCVD coatings from **(a)** CoD-4-BrPP and **(b)** CoDPFPP.

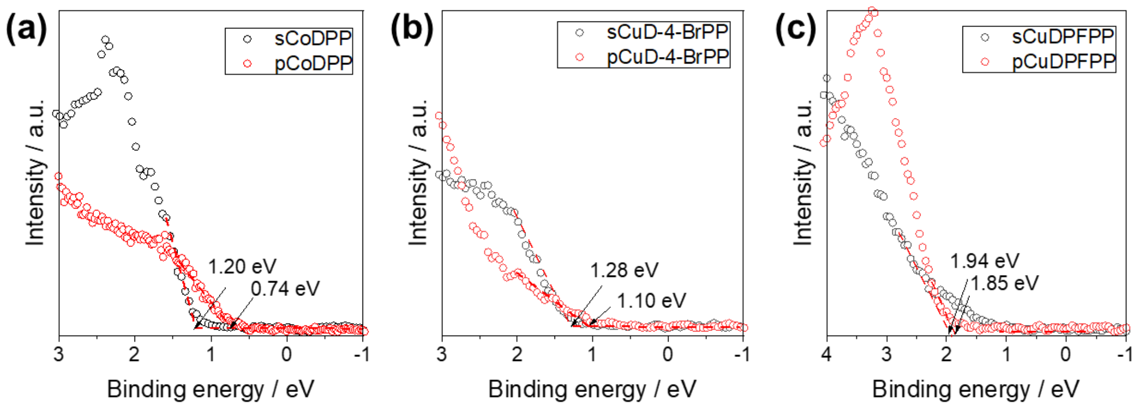

Figure S12. XPS spectra on the valence band region of the reference (sublimed) (black dots) and oCVD (red dots) coatings.

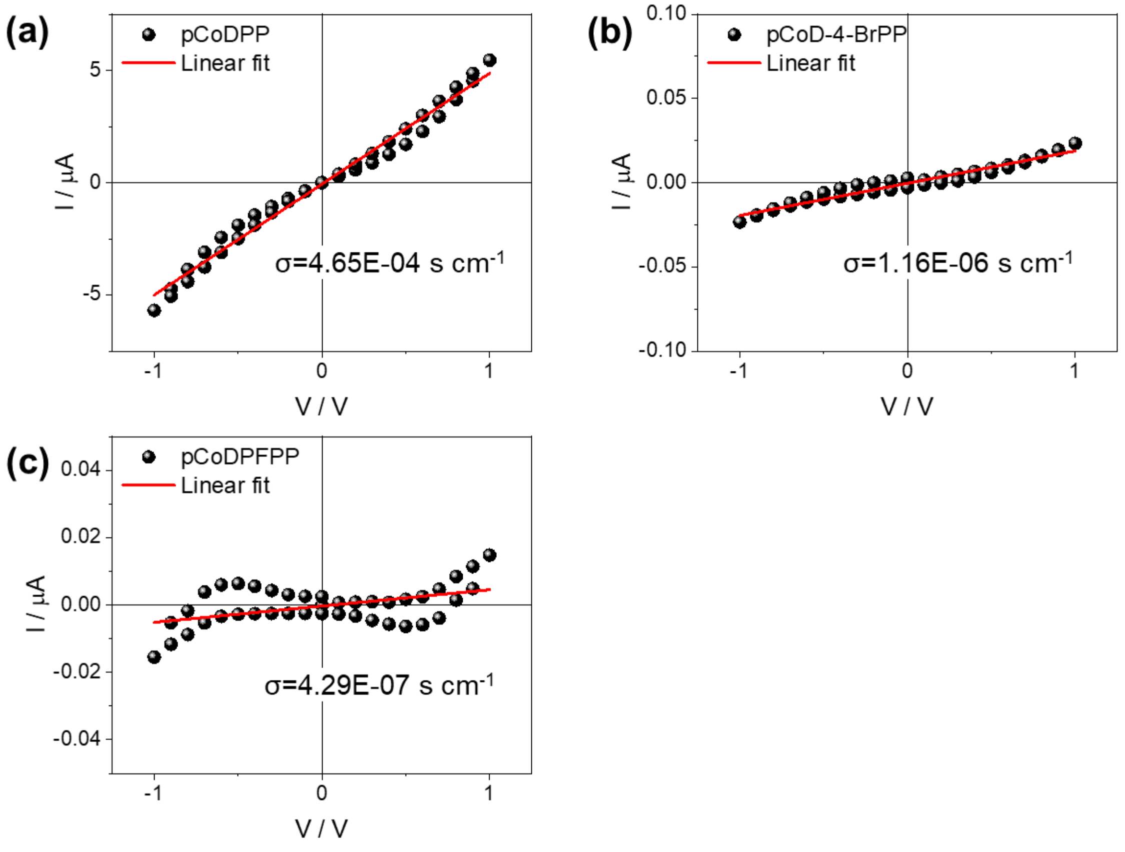

Figure S13. Lateral conductivity of the oCVD thin films coated on OFET substrates, calculated from the 2-points probe method (length of the chip= 2.5 µm)

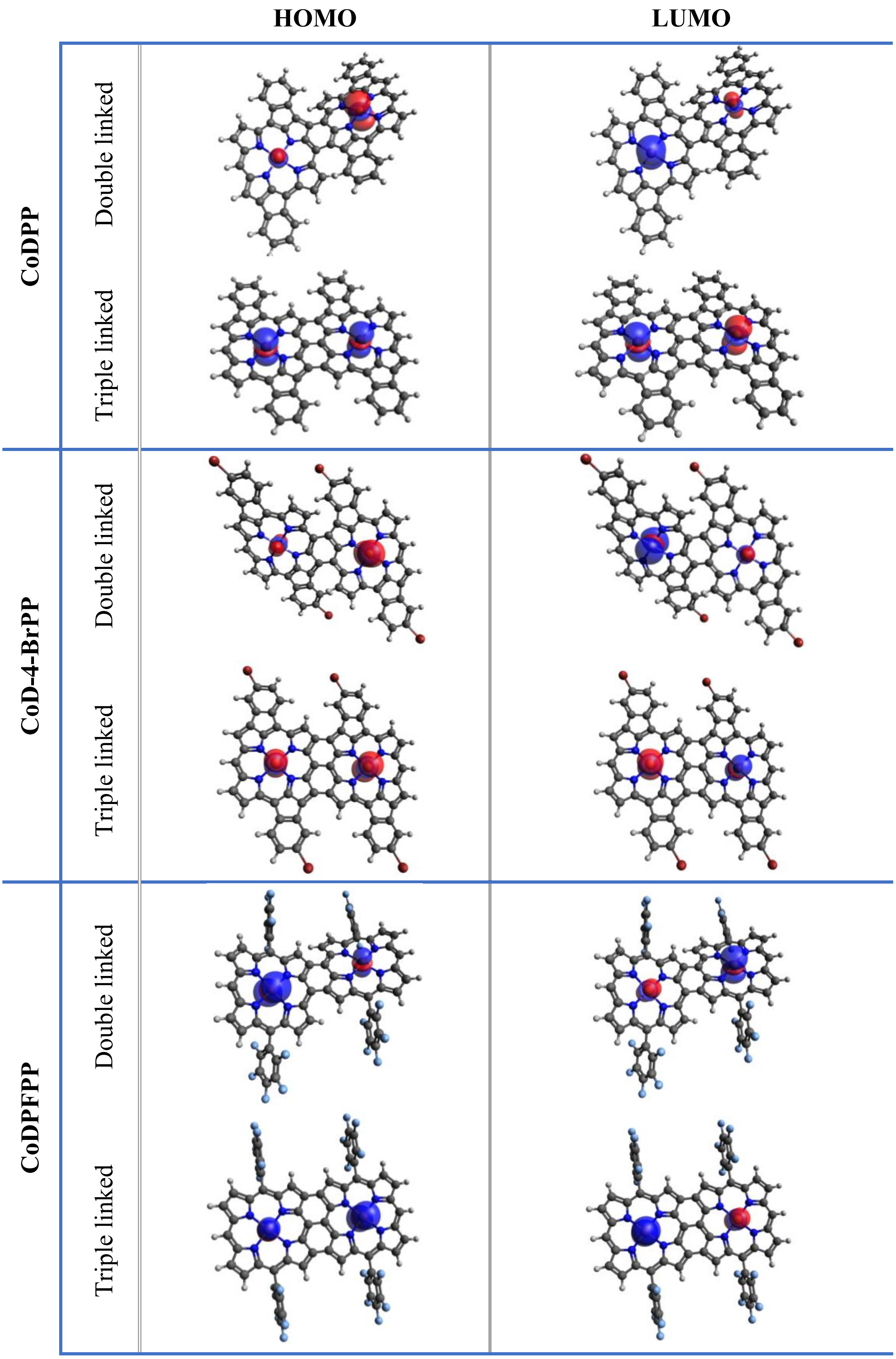

Figure S14. Optimized structures of doubly and triply fused cobalt porphyrin dimers, showing the distribution of frontier molecular orbitals.

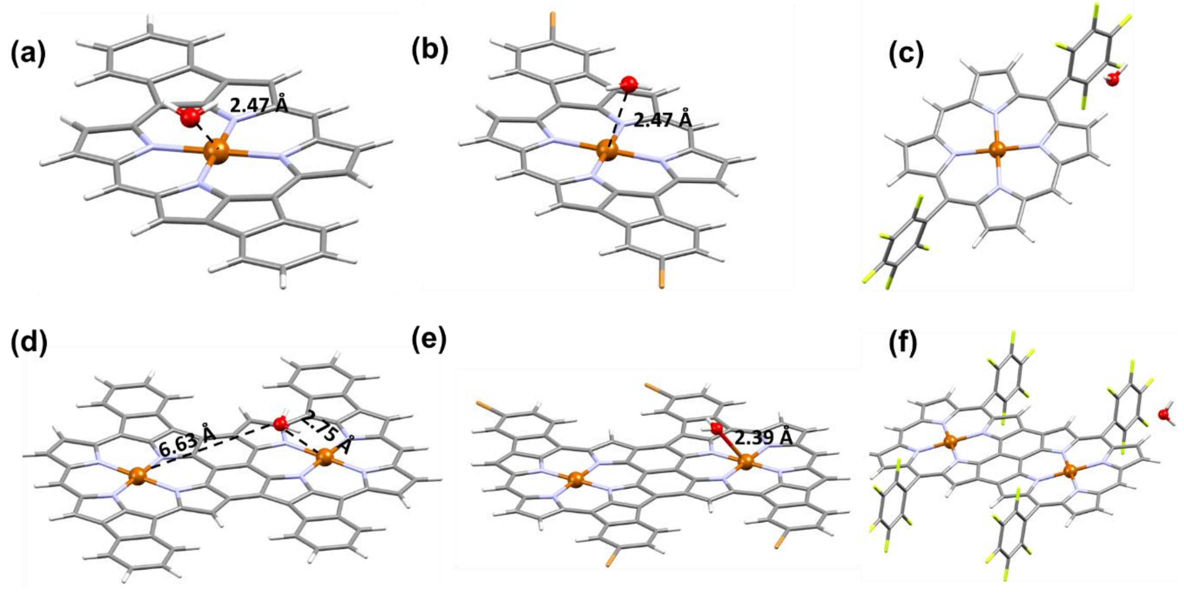

Figure S15. Water docking distance calculations showing the interaction of water molecule with the optimized **a-c)** monomers and **d-f)** triply fused dimers, considering intramolecular cyclization.

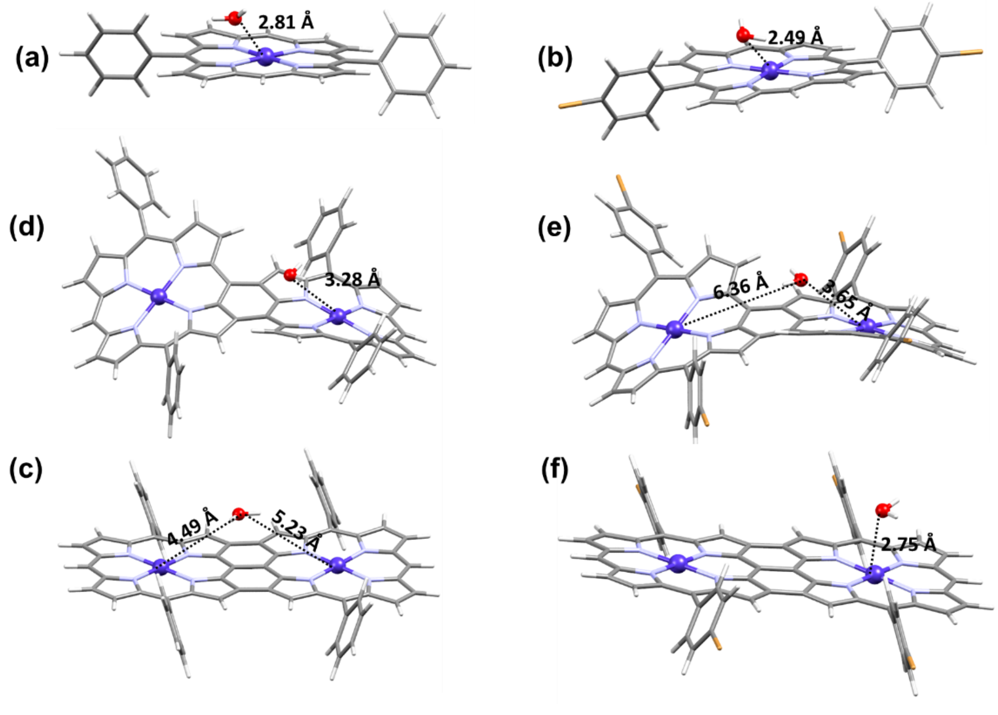

Figure S16. Water docking distance calculations showing the interaction of water molecule with the optimized **a-b)** monomers, **c-d)** doubly and **e-f)** triply fused dimers, without considering intramolecular cyclization.

Table S3. Coordinates for doubly linked CoDPP dimer.

	X	Y	Z
C	-3.31753365088313	-3.80824127675416	-0.59260074593947
C	-2.46370503973347	-4.85123238909562	-0.32027696809278
C	-1.16628255232980	-4.46862253194508	-0.81960335000648
N	-1.22021216948852	-3.19539254447281	-1.40087733124455
C	-2.53813799359604	-2.78477370343317	-1.24845171293618
C	-1.62191947817255	1.51685037258746	-2.91525623569410
C	-2.73810363738971	0.85128787193918	-2.44541324827628
C	-2.30240007909349	-0.51507667802028	-2.15528984727965
N	-0.94909304180100	-0.67717137758611	-2.46358217899171
C	-0.56218273884923	0.53877085679737	-2.91581612833836
C	-3.06490348830644	-1.53716003000547	-1.60895935109854
C	3.76304721795269	-0.58941320634860	-4.20960619267822
C	3.03125537490267	0.57367977057104	-4.13803415630483
C	1.75045031484878	0.22632511324105	-3.57908342384267
N	1.71009874017165	-1.14181861106626	-3.26174922771429
C	2.93182433979242	-1.64467043423042	-3.68334364190218
C	0.63927890048306	1.05775662677534	-3.41127810499970
C	2.25797062063604	-5.68638123361076	-1.36353722760637
C	3.28885911829717	-5.09365567322290	-2.11227200653123
C	2.72343385944729	-3.82412500924213	-2.60075060997743
N	1.45838673219912	-3.60331406998013	-2.09672589402462
C	1.18053264615469	-4.73164647953835	-1.38528847267906
C	0.01673616807243	-5.21828849236245	-0.78546777886979
C	3.38519138693488	-2.97669817155035	-3.50413284342832
Co	0.23960950918468	-2.16214060777780	-2.32006833000135
C	0.35517210437836	2.48616142029879	-3.72759867935542
C	0.35585306248869	-6.58974253968417	-0.31698911696589
C	1.16574462005980	3.50800635047264	-4.23279886353680

C	0.61501082497376	4.79299266365241	-4.43824555519905
C	-0.73137558694342	5.05557239796228	-4.14441516220615
C	-1.56577885560492	4.03661807431985	-3.63412848890238
C	-1.03345139632902	2.76207857394165	-3.42484981463273
C	-0.40998585908826	-7.55894100295202	0.33596925780459
C	0.14888280420977	-8.83155449047535	0.59415252645522
C	1.45581359581453	-9.13209897680400	0.18799898021186
C	2.25117043200572	-8.16062004831426	-0.45930363841897
C	1.72419754857646	-6.88528703384721	-0.69503090990617
H	-4.38621114214452	-3.72306126682153	-0.36224353961956
H	-2.69604017797662	-5.79859197489735	0.17504526562271
H	-3.76092619674186	1.21513268694396	-2.28859035113486
H	-4.12879910667379	-1.35225378620486	-1.40482874346492
H	4.79941225958533	-0.71715789725954	-4.53533118924289
H	3.34529365120075	1.58048998005101	-4.42918358553213
H	2.22311788040326	3.32859215573390	-4.47060898580872
H	1.25453044674070	5.59620823886314	-4.83441567093598
H	-2.62180635298342	4.24268062831054	-3.40392065214302
H	-1.44352930512171	-7.35033485997950	0.64388514103581
H	-0.45664005092018	-9.59386703765351	1.10721241245953
H	3.27219872671327	-8.40869397081404	-0.77901934294318
C	5.17302464972526	-6.84300029382735	-0.45678912239020
C	6.20803632970409	-7.67052078053951	-0.08616210896166
C	7.10281171931852	-7.74820329321036	-1.21175612662824
N	6.64395604362263	-6.93142658383201	-2.25871346466421
C	5.44268979219220	-6.40652356697539	-1.80523025874121
C	5.31751732102048	-3.24524535746486	-5.29293562546804
C	4.55662870905850	-3.54078489112294	-4.14907463414715
C	5.17771467389865	-4.74892662801101	-3.57922317703348

N	6.31285211332656	-5.11262763278065	-4.27363372012285
C	6.36976230028328	-4.22757488497311	-5.30803627892398
C	4.64211192911010	-5.45739237129251	-2.49131363285542
C	9.80581852674666	-6.32275934446824	-7.65670979051702
C	8.99700696866848	-5.22966737562322	-7.86176788249866
C	8.15406171784872	-5.11133023989055	-6.69795894902520
N	8.43864421027814	-6.13009351315645	-5.78006139094248
C	9.46334534232689	-6.86449642093758	-6.36274837339620
C	7.15686062892992	-4.15675206179501	-6.45974187220952
C	10.03440836870325	-9.52536424882735	-2.52655038450357
C	10.61696580379011	-9.44884064075177	-3.77731790717182
C	9.88176798291113	-8.41303099055713	-4.50370239553385
N	8.85905196576877	-7.88602000273081	-3.71061033370156
C	8.96324590541616	-8.55991455573214	-2.54111201197711
C	8.24099898148125	-8.54937874213560	-1.34247561838991
C	10.14142353727379	-7.94567805886972	-5.78376912100222
Co	7.55525852367337	-6.52778621750235	-4.01794587905140
C	6.59411441762988	-3.03231700525022	-7.25603917612453
C	8.90679857377179	-9.55642011546663	-0.46846565314283
C	6.95898348049493	-2.50783451315770	-8.49858936447700
C	6.18686902426727	-1.47146031327267	-9.07146602528717
C	5.05340125943915	-0.97897509730011	-8.41099604950751
C	4.67765014235396	-1.48561316657533	-7.14704106239770
C	5.44918036572682	-2.49016759981253	-6.55081304226741
C	8.64548410416070	-9.97151826946593	0.84139364923309
C	9.45708578436955	-10.96817321795893	1.42850292304251
C	10.51798799737993	-11.54607444726188	0.71537993142272
C	10.79753569764090	-11.14003598861610	-0.60819993660235
C	10.00364470710511	-10.15455885306416	-1.20000980780434

H	4.32696345619424	-6.50582031367692	0.14902311926011
H	6.35241232472642	-8.17131703388325	0.87582326425584
H	10.58191645259492	-6.73078535983763	-8.31517365440856
H	8.97304130089490	-4.56395448316452	-8.72952818387179
H	11.46087002511523	-10.00999244097497	-4.19691415213647
H	10.94882353285229	-8.41361436038393	-6.36417584825221
H	7.83185173045364	-2.89597138846557	-9.04065225515091
H	6.47490864117619	-1.06030316449779	-10.05073251533082
H	3.78357127475373	-1.09686744106832	-6.64138971664135
H	7.82155004691624	-9.53413964809515	1.42153241887908
H	9.25064451790193	-11.29283163255974	2.45955294690609
H	11.62995361597549	-11.59329905335291	-1.16703917148741
H	-1.14177392152925	6.06281424171060	-4.31216410901296
H	4.44643283970382	-0.18832274837239	-8.87744938497403
H	1.87174191783090	-10.13394124325214	0.37352369812699
H	11.13811765896515	-12.32164302242664	1.18976093242550

Table S4. Coordinates for triply linked CoDPP dimer.

	**X**	**Y**	**Z**
C	-2.09812719525494	-4.51562656361948	2.18911263415724
C	-1.09753637300063	-5.46676337000903	2.24383090049576
C	-0.11012980241651	-5.04568553834705	1.28077004872114
N	-0.44012014401010	-3.89677815308007	0.63820695971840
C	-1.67613270651933	-3.54124327973036	1.18375167669627
C	-1.88829614865736	0.30521178787525	-1.54098255884847
C	-2.63678121281212	-0.33015324667356	-0.57819257524122
C	-1.90311161118269	-1.50932899534234	-0.18289106335210
N	-0.71179048095313	-1.60737702836383	-0.89156430995865
C	-0.69884679056708	-0.48574029255858	-1.73314698194746
C	-2.36736669604597	-2.40444145527424	0.78835479182862

C	3.43623557620824	-1.67442393108839	-3.57387817794540
C	2.43464369348953	-0.69969939784790	-3.63601255744110
C	1.44154921351332	-1.10943308978051	-2.67597351153842
N	1.75045591010445	-2.26309154550498	-2.02193752325918
C	2.96968285773465	-2.62682632321123	-2.55664119364620
C	0.35950228686466	-0.23139991251099	-2.61626725974959
C	3.21957886876492	-6.46879658885818	0.14455345439294
C	3.97521405649920	-5.83093674243700	-0.83321564345105
C	3.21600087344941	-4.64816223251735	-1.21220577106391
N	2.03827613698760	-4.55209628868299	-0.50347132236200
C	2.03656959619758	-5.68060309867197	0.34043161533330
C	0.97713681864542	-5.92476354664717	1.22272415243656
C	3.69560807496149	-3.75820528951691	-2.19351922081194
Co	0.65612624506299	-3.07909986990848	-0.69289811822956
C	0.66367081938269	0.83811754045513	-3.61014088677048
C	0.68286820105975	-6.99375986806503	2.21609939767988
C	1.94087037816188	0.54211330764366	-4.23167355203434
C	2.46637168605192	1.39402185867999	-5.20746810443735
C	1.74011940448311	2.54605663578957	-5.58255798686076
C	0.50541540050738	2.83446501456022	-4.98415698510910
C	-0.03937107993390	1.98225990690611	-3.99479225709061
C	-0.59157853316075	-6.70929333514412	2.84062369407434
C	-1.10849355885232	-7.56241947571943	3.81827952018877
C	-0.37112673387443	-8.70817425290504	4.19132028808902
C	0.86463677134796	-8.98756894001536	3.58912907733833
C	1.39878032460298	-8.13286554024674	2.59879547907705
H	-3.03635605183904	-4.44622749566450	2.75301456577643
H	-2.12357723226184	1.23139111048481	-2.07434751174719
H	-3.60700229671215	-0.03453241466584	-0.16152776957815

H	-3.33532650069643	-2.18705128700823	1.26131986152248
H	3.46103615371815	-7.39456870417585	0.67408979204872
H	3.43506925436035	1.16982187381624	-5.67738529486743
H	-0.05067541404375	3.73511300938279	-5.28507046106819
H	-1.00843657429310	2.23173369348258	-3.54115895479679
H	-2.07762833753754	-7.34413965252175	4.29137905131974
H	1.42859306030744	-9.88349526541459	3.88960723979936
H	2.36876863306264	-8.37399996607691	2.14280422540428
C	5.23691567599742	-6.09572440971687	-1.47255468419329
C	6.23849494325914	-7.07046495992246	-1.41043859417867
C	7.23165847858583	-6.66064870775600	-2.37036758772170
N	6.92277601369592	-5.50697037214622	-3.02437727500898
C	5.70352432899249	-5.14326129367395	-2.48970843538017
C	5.45344627903461	-1.30149505215771	-5.19117783338397
C	4.69790873595061	-1.93924466538241	-4.21325995974383
C	5.45718227033403	-3.12195155131470	-3.83418080103280
N	6.63485933777469	-3.21807518371605	-4.54298802385676
C	6.63648011791170	-2.08966053537815	-5.38701576737381
C	4.97760546541982	-4.01187350789509	-2.85281839870857
C	10.77173538827496	-3.25399369878001	-7.23485763775403
C	9.77080832693932	-2.30323839561305	-7.29007948689205
C	8.78331225123457	-2.72442511863299	-6.32715945568462
N	9.11352561571838	-3.87308112956360	-5.68426020918531
C	10.34982322078461	-4.22828641446306	-6.22937720170247
C	7.69588248001170	-1.84553057721545	-6.26935503206073
C	10.56237176154724	-8.07428723950091	-3.50403803148491
C	11.31103701456522	-7.43871604895091	-4.46655211475679
C	10.57709061655039	-6.25985549585698	-4.86227403141435
N	9.38540993678398	-6.16220472525076	-4.15414317818607

C	9.37254119704819	-7.28377041080999	-3.31245688122290
C	8.31392723718781	-7.53843155141754	-2.42974538583030
C	11.04136486779146	-5.36473789957160	-5.83350798197183
Co	8.01711710455982	-4.69097723690700	-4.35342366154258
C	7.99006665250798	-0.77662546797989	-7.26285281305690
C	8.00975783875700	-8.60795741887117	-1.43588185458456
C	9.26466654184729	-1.06091494338832	-7.88714519472896
C	9.78154532683346	-0.20782540999220	-8.86485259654392
C	9.04399366367138	0.93772057934093	-9.23816825827742
C	7.80808165355508	1.21694334066414	-8.63620179605129
C	7.27397037949162	0.36227198599493	-7.64582344441064
C	6.73238739248568	-8.31214788838522	-0.81460606765506
C	6.20685790653287	-9.16409361275981	0.16114108476988
C	6.93325033644211	-10.31597260033717	0.53643741063638
C	8.16811221454640	-10.60419992441024	-0.06172501152088
C	8.71292677260742	-9.75195883589261	-1.05104306667536
H	5.21194056250640	-0.37577908014833	-5.72079038328586
H	11.71015574436076	-3.32316936942682	-7.79846788351470
H	10.79777645797937	-9.00031405487707	-2.97046347316690
H	12.28155668899250	-7.73399412816119	-4.88276410488401
H	12.00959250478977	-5.58182979297843	-6.30606086538204
H	10.75079621990452	-0.42597002118171	-9.33777648984137
H	7.24398088042493	2.11270640032321	-8.93689547275359
H	5.23803027530049	-8.94004318864055	0.63086161691227
H	8.72430616928920	-11.50473344686584	0.23934045643968
H	9.68210940133081	-10.00130031788452	-1.50449952890470
H	9.44342942806706	1.61555728709975	-10.00762275960039
H	2.14809426634083	3.22096174740447	-6.34997724187313
H	6.52525782110978	-10.99090235500710	1.30382563705007

H	-0.77058963577276	-9.38603838461901	4.96073656125399
H	6.30385904923928	0.60326132130309	-7.19001768297099

Table S5. Coordinates for doubly linked CoD-4-BrPP dimer.

	**X**	**Y**	**Z**
C	-3.27059128142356	-3.79924873588286	-0.58217710455009
C	-2.41984289231000	-4.83435957215408	-0.31033502799457
C	-1.12754316033695	-4.45576904120911	-0.80704745514711
N	-1.18486202996257	-3.18559162239396	-1.39045798739517
C	-2.50074644786880	-2.77817844059175	-1.23858606327565
C	-1.61639287623159	1.48748001996488	-2.95421092090562
C	-2.72087002494616	0.82811835115478	-2.47041122750556
C	-2.27997382943523	-0.52531831148131	-2.15983775754924
N	-0.92874920662410	-0.68217696476589	-2.46941979724521
C	-0.55214490980729	0.52369531622189	-2.94215058819563
C	-3.02882855288235	-1.54066687591862	-1.60285045500761
C	3.77379532053557	-0.57905006060854	-4.18673373826666
C	3.03443505419457	0.57064063167091	-4.14201680440793
C	1.75554787032273	0.22465448747602	-3.59447709666128
N	1.72596926386708	-1.13774782772156	-3.25719759348590
C	2.95352526474473	-1.63354061063817	-3.66024966009452
C	0.63928332861648	1.03907900659601	-3.44742487204077
C	2.29148218452432	-5.65047853363758	-1.33555632382873
C	3.31751776749349	-5.06170593549114	-2.08063299713876
C	2.75410069373007	-3.79949965829737	-2.57132112174636
N	1.48968903227052	-3.58279737898397	-2.07250971787051
C	1.21414292494478	-4.70584190665926	-1.35990670847347
C	0.05391630755916	-5.19301816200067	-0.76903758031853
C	3.41039563359335	-2.95496004515050	-3.46931546598989
Co	0.26553346928469	-2.15500220079084	-2.31185665338888

C	0.34271900491629	2.45158740469351	-3.79268518002222
C	0.39465327950290	-6.55729482026621	-0.29980432531326
C	1.13152762599326	3.46397916239037	-4.32739208365363
C	0.57682151058473	4.73331530014661	-4.56190103043001
C	-0.76212079243131	4.96995573939307	-4.25972992678085
C	-1.59140083936858	3.97364850229618	-3.72183168897639
C	-1.04020482579632	2.72094647932351	-3.48999479834150
C	-0.35997275282037	-7.52765072050508	0.34657739782051
C	0.19662982039725	-8.79106824211196	0.61357907152793
C	1.49895975501611	-9.06315282275901	0.20774840517365
C	2.29615397116599	-8.10721530754750	-0.43993444772760
C	1.75932121773857	-6.84560292592868	-0.67190044319270
H	-4.32951938136856	-3.71859192882920	-0.35239489061990
H	-2.65235883716069	-5.77191271195216	0.18153924439619
H	-3.73562722042945	1.18629690457866	-2.31811500340966
H	-4.08431603522963	-1.36230264274472	-1.39873993465714
H	4.80528094014302	-0.69766828871862	-4.49808529803729
H	3.34303262213003	1.56528630408967	-4.44298226427857
H	2.17867976866403	3.29725520810455	-4.57447001630078
H	1.18930340466952	5.53031752900273	-4.98034604424017
H	-2.63606636476021	4.18433027637628	-3.49738454856329
H	-1.38660089480029	-7.33408735613435	0.65299022015210
H	-0.38956128845089	-9.55418459198517	1.12284848837035
H	3.30840314799678	-8.35761693349400	-0.74882571557112
C	5.18859205133007	-6.82216670029055	-0.44166316515048
C	6.20206808598161	-7.66878134560852	-0.08578530917514
C	7.08828241165906	-7.75581799420731	-1.20923541174584
N	6.64070089491322	-6.92059424187901	-2.24484189791372
C	5.45268618219137	-6.38126863340211	-1.78253898901188

C	5.33848202384615	-3.22124949342293	-5.24397754138955
C	4.57950044930711	-3.51529274560620	-4.10731926002282
C	5.19476777965621	-4.72025687303116	-3.54065755990830
N	6.32494177554200	-5.08500929540455	-4.23649953100629
C	6.38438542809965	-4.20041189747424	-5.26549210525781
C	4.66363915598348	-5.42654556442469	-2.45923722691560
C	9.79482881971766	-6.29334287091778	-7.61684589087781
C	8.99248385205002	-5.20467401246095	-7.81763913488152
C	8.15120793887724	-5.08561674923024	-6.66082698555045
N	8.43360898899006	-6.10705312383085	-5.74793723503236
C	9.45457520076278	-6.84016041467087	-6.33179170887229
C	7.16364623672940	-4.13436168545996	-6.41486972801839
C	9.98690487940008	-9.54198883553954	-2.54506079177771
C	10.57571939459337	-9.45208786597904	-3.78359791329563
C	9.85971687591121	-8.40240341527896	-4.49657344747822
N	8.84073397441516	-7.87861271768872	-3.70054864189341
C	8.93045129662369	-8.56961942521729	-2.54569103241621
C	8.20537538486061	-8.56926083004745	-1.35621123509320
C	10.12377358046500	-7.92210519098555	-5.76202762326992
Co	7.55173757132912	-6.50997298722565	-3.99446906591246
C	6.60342181009528	-3.01274235322925	-7.20576066722498
C	8.84948975306942	-9.59803340411936	-0.50218862655018
C	6.95576453662955	-2.48939058548982	-8.44296175061476
C	6.19354390154782	-1.45436243037868	-9.01287122738865
C	5.07699140239200	-0.97643968140624	-8.33471911014115
C	4.69386042119592	-1.47260802159321	-7.07928837484802
C	5.46785668492675	-2.47116269478339	-6.49819995569916
C	8.57598521967100	-10.04054708035773	0.78709924053680
C	9.36271692460105	-11.05311390303415	1.36120722126432

C	10.41172026951571	-11.61034867532674	0.63371158627714
C	10.71732465808082	-11.19306303403326	-0.67098021796256
C	9.93904363492299	-10.19159801495650	-1.23501552981324
H	4.36005255479345	-6.47966704588341	0.16761199014866
H	6.33629285831774	-8.17424466301431	0.86376537019473
H	10.56299383101932	-6.69500325015700	-8.27199626167473
H	8.97254065048662	-4.54511302798575	-8.67758185204148
H	11.40849360515276	-10.01105318448222	-4.20222121593607
H	10.92274495376220	-8.38343666574742	-6.34195966602205
H	7.81596429002223	-2.86924596960760	-8.99140908712951
H	6.46625381421106	-1.03826811801418	-9.98125612085095
H	3.81015159612443	-1.07935979652937	-6.58216557700856
H	7.75837079813594	-9.61975427672453	1.36991419816063
H	9.15388557246277	-11.40382701401901	2.37057138619686
H	11.54191137631075	-11.64718580084423	-1.21864637409830
Br	-1.50079445570748	6.70020829867465	-4.58859618596158
Br	4.02110698081467	0.40779928515930	-9.12213002069747
Br	2.24628963920217	-10.78932696102347	0.54400199804878
Br	11.47046674485208	-12.98704246793432	1.42796879386196

Table S6. Coordinates for triply linked CoD-4-BrPP dimer.

	X	Y	Z
C	-2.08860220206574	-4.53107557857885	2.19458670627900
C	-1.08757903216328	-5.48203942102307	2.24793068285599
C	-0.10066513373103	-5.06165018051438	1.28493539291330
N	-0.43097710022987	-3.91297394799702	0.64334745741984
C	-1.66677167373503	-3.55697871986576	1.18938702843797
C	-1.88010231132144	0.29047437603423	-1.53492905374402
C	-2.62785255366245	-0.34515975742655	-0.57205113470680
C	-1.89368960577333	-1.52449583131347	-0.17733708327128

N	-0.70245009708971	-1.62212220204795	-0.88680336905919
C	-0.69060482392429	-0.50017532343849	-1.72788985057420
C	-2.35756854049950	-2.41957592630204	0.79386733772881
C	3.44547073211122	-1.68983665897476	-3.56853761934791
C	2.44326325510333	-0.71638692807991	-3.62959201243264
C	1.44992639810736	-1.12451147822594	-2.67067561357279
N	1.75933894316194	-2.27789094554134	-2.01722292769822
C	2.97857539323239	-2.64168680034789	-2.55175305294440
C	0.36762480543295	-0.24539802922111	-2.61152587575579
C	3.22920708910942	-6.48401106598181	0.14790958133644
C	3.98438767221474	-5.84510571495656	-0.82957617001391
C	3.22514857974080	-4.66277371210796	-1.20817006273760
N	2.04733164932004	-4.56736521841902	-0.49942425156582
C	2.04624498594273	-5.69616954945028	0.34369467438351
C	0.98711898559056	-5.94120205683791	1.22611688813249
C	3.70479539640463	-3.77282039534514	-2.18910983932636
Co	0.66511958989781	-3.09457698985476	-0.68804763580472
C	0.67220691989485	0.82224522692311	-3.60456085818366
C	0.69467532638426	-7.00885569477545	2.21726770411017
C	1.94949161210670	0.52424208320582	-4.22477388567524
C	2.48563837183553	1.36592608934437	-5.20108837479377
C	1.75340877942466	2.51554038960859	-5.57145687103512
C	0.51733969862906	2.82190136577455	-4.98487987452489
C	-0.02387113037180	1.96854297103744	-3.99592604454042
C	-0.57948685078245	-6.72454696015718	2.84305125704794
C	-1.10268603669938	-7.57111231734355	3.82126295700888
C	-0.35492080002231	-8.71355053740818	4.18489532130091
C	0.88152031206182	-9.00757844612316	3.59121270823608
C	1.40773399752256	-8.14917818758735	2.60197618357591

H	-3.02621555400151	-4.46240819270930	2.75947952677145
H	-2.11666334998589	1.21689435625234	-2.06747818609145
H	-3.59787295933136	-0.04958563671473	-0.15501663838927
H	-3.32531793523598	-2.20185616262558	1.26706041103417
H	3.47058684701864	-7.40995526501255	0.67730676595548
H	3.45218892882625	1.14660140624706	-5.67357971988136
H	-0.99328596918918	2.22625537129063	-3.54796813371355
H	-2.06891280722893	-7.36130201463608	4.30030241398261
H	1.43745469956658	-9.90438758611753	3.89743152712741
H	2.37757898816493	-8.39781865978494	2.15015146902798
C	5.24571033911110	-6.10843941607799	-1.46920478077944
C	6.24777379901621	-7.08200222154331	-1.40790633841830
C	7.24126105329027	-6.67406662078137	-2.36673315272436
N	6.93207688204597	-5.52072778979187	-3.02041684148513
C	5.71276848844012	-5.15671963703347	-2.48603072644866
C	5.46206036526515	-1.31426977898940	-5.18563831582771
C	4.70681230181911	-1.95315773983926	-4.20816895729384
C	5.46607572911730	-3.13553174153022	-3.82957529681916
N	6.64379585248664	-3.23089984616918	-4.53831566633358
C	6.64502302222452	-2.10212910143618	-5.38140217530468
C	4.98653193854460	-4.02559273098410	-2.84866242376412
C	10.78035039630680	-3.26789615141515	-7.23111260977728
C	9.77930494042563	-2.31701226655313	-7.28482102330228
C	8.79210212385594	-2.73723514397532	-6.32204021054678
N	9.12230268127376	-3.88588516864620	-5.68021111239283
C	10.35830783809436	-4.24185195356353	-6.22587991773468
C	7.70439545477002	-1.85744696058767	-6.26362024835027
C	10.57186729894965	-8.08893316785345	-3.50103527868766
C	11.31983711698844	-7.45339219862698	-4.46381652599480

C	10.58564555980346	-6.27426024725196	-4.85902240492301
N	9.39414425351453	-6.17674373543039	-4.14998227706952
C	9.38215834855976	-7.29840375545934	-3.30866779328310
C	8.32361259822356	-7.55310377662477	-2.42533747039099
C	11.04933451007570	-5.37906130942954	-5.83017050561870
Co	8.02620966575701	-4.70431451097366	-4.34943072796315
C	7.99729601002222	-0.79001935835320	-7.25479184560495
C	8.01891960824527	-8.62055775379122	-1.43209788655329
C	9.27149581764851	-1.07449087555227	-7.88020135796554
C	9.79509816744345	-0.22813802277882	-8.85836352039049
C	9.04791579292076	0.91459000691487	-9.22264564728342
C	7.81119067080820	1.20868148297712	-8.62903069267104
C	7.28465009657748	0.35046894846773	-7.63997094130053
C	6.74139183401251	-8.32240529846257	-0.81225517907311
C	6.20515631759175	-9.16395686483440	0.16412088059756
C	6.93771879108749	-10.31321609393286	0.53463246644274
C	8.17377373560213	-10.61981558200365	-0.05126879074161
C	8.71514198729644	-9.76659858390511	-1.04044767244228
H	5.22067469920550	-0.38829625048336	-5.71501013063834
H	11.71810883886370	-3.33653479486604	-7.79576187282635
H	10.80823492828224	-9.01515863194627	-2.96807568543124
H	12.29005090220362	-7.74888618593193	-4.88046319039992
H	12.01722713350771	-5.59633488306046	-6.30328749716422
H	10.76151794432580	-0.43864475530974	-9.33675197504311
H	5.23856024852329	-8.94518664885817	0.63672885699122
H	8.71881589139043	-11.52183263215299	0.25924488607447
H	9.68470396357827	-10.02483613580483	-1.48775374088991
Br	9.74582828415777	2.08328862461863	-10.55812309851967
Br	2.47631485638892	3.67235981979829	-6.90526989279355

Br	6.21301952591506	-11.46820189006898	1.86906493250940
Br	-1.05272980500084	-9.88156205372905	5.52086419844211
H	6.31476311444535	0.59874661550479	-7.18800844273193
H	-0.02782167552477	3.72404592972528	-5.29483338358352
H	7.25526827276057	2.10552329551641	-8.93520085105719

Table S7. Coordinates for doubly linked CoDPFPP dimer.

	X	Y	Z
C	-0.88181511423024	-3.31105273390503	6.43589670164440
C	-2.01486017218586	-3.19377994293198	5.68632698494020
C	-1.61607493109538	-2.65912701722001	4.40799832680858
N	-0.23988086193532	-2.49848906310447	4.35755662404402
C	0.21648061230609	-2.90048063732914	5.60041649813644
C	4.67750759465905	-3.02227178854087	4.23751319854574
C	3.97083325165472	-3.14970819646300	5.39685818135149
C	2.60154411698131	-2.82781893443202	5.08858145907279
N	2.47608523442746	-2.45778485951915	3.75888410260883
C	3.74717867425647	-2.57913323522191	3.22819000975179
C	1.54788607275311	-2.99374466529846	5.96821244913115
C	2.57334742264922	-0.37079236698777	-0.71651792899047
C	3.62577438383211	-1.09971115507561	-0.24465276138931
C	3.23531331247836	-1.61399474785724	1.04090226591690
N	1.92010517781670	-1.26712025367538	1.31942251968749
C	1.51829929603753	-0.47567187678386	0.25710867305146
C	4.10148861422165	-2.25346825999270	1.92170711467669
C	-2.98637649325811	-0.96375903083854	1.33851569763357
C	-2.21881894814639	-0.25710037616671	0.43443459292743
C	-0.84320313993565	-0.48273025554862	0.83292679525946
N	-0.76996113250256	-1.30540488201994	1.93261723149490
C	-2.08867532186105	-1.58631007404960	2.26403389219234

C	-2.49924054556832	-2.27170131322968	3.40613561916594
C	0.22307141143396	0.03998111150760	0.09706436045849
Co	0.84701472666423	-1.87390769080645	2.84918692881891
C	5.50160275916618	-2.51369028852163	1.48605304251577
C	-3.95751396946271	-2.50341081761372	3.59463191835348
C	6.45989197312678	-1.49409005333803	1.44756984197281
C	7.77610184162724	-1.73139693603412	1.04683938988889
C	8.15752190416415	-3.02271878981401	0.67380144271869
C	7.22449652990717	-4.06208706645448	0.70413961228020
C	5.91452055502400	-3.79632996328400	1.10822479914536
C	-4.62838012912222	-3.50499310091991	2.88314207785799
C	-5.99976156711335	-3.72308105784271	3.02754153634746
C	-6.73330276746563	-2.92201649940146	3.90625758650544
C	-6.09315572167987	-1.91199078521571	4.62882606435448
C	-4.72094019635754	-1.71426751917412	4.46346251059869
C	-4.82450493729187	-0.39980752690659	-1.16291887252164
C	-5.84832803952158	-0.06705136756716	-2.00150057931273
C	-5.49791773917752	1.18785564110614	-2.61147377720964
N	-4.23143126598703	1.58442985682021	-2.20479530803166
C	-3.82370052092732	0.62682591837894	-1.29220822772604
C	0.63175624268502	1.94677979551516	-1.61154599184803
C	-0.12533747764265	1.05996711386136	-0.87227837035531
C	-1.50476060000402	1.35040260900502	-1.21107278501330
N	-1.58922168997041	2.36928905869857	-2.13071038528253
C	-0.27714374472697	2.75860766997641	-2.36341583541781
C	-2.55779401028652	0.59636202725312	-0.68719404614870
C	-1.61961124163572	6.69652076530620	-4.49066874107850
C	-0.45296736977161	6.03462213712412	-4.24472874059537
C	-0.80709925005465	4.77693987993885	-3.63488775840101

N	-2.18516698248249	4.64849780080037	-3.55458041069053
C	-2.68965980830376	5.82322642235407	-4.08381906767231
C	0.10774839562670	3.86588074924385	-3.11757923277113
C	-7.02817149590300	4.13849676760273	-4.40879361196035
C	-6.39256782028622	5.33288591030128	-4.58063132840539
C	-5.03569749023894	5.15224731967842	-4.13284400630198
N	-4.85230326272160	3.86928772933318	-3.64259649200536
C	-6.07194097589334	3.23948467907105	-3.81058806728515
C	-6.36452943231887	1.94428342977556	-3.39322351809868
C	-4.03176947838676	6.08907140791307	-4.29642681583917
Co	-3.21599210296850	3.12589525790836	-2.87562686194150
C	1.56409662097820	4.12049066783422	-3.28990136987336
C	-7.70590325205258	1.38431443716780	-3.71942229921215
C	2.32389576899664	3.36710601190589	-4.19281324499698
C	3.69530307578546	3.57044633186743	-4.35621162335282
C	4.33799098055211	4.55374084157353	-3.60007703666569
C	3.60794265111677	5.32059328812644	-2.68838758390669
C	2.23781384134312	5.09400589236122	-2.54276935095130
C	-8.76529590812834	1.43386069371834	-2.80657897504800
C	-10.02696958775970	0.91905838176014	-3.11101548599796
C	-10.24647233464805	0.33564568943373	-4.36161490649661
C	-9.20835224631117	0.27168933644361	-5.29433062501216
C	-7.95676612645096	0.79511972393572	-4.96339882577392
H	-0.77971309829864	-3.66710135861908	7.45711680527743
H	-3.03618917396278	-3.42571550307969	5.97072248388798
H	5.73671696281973	-3.19166404790842	4.07211661935030
H	4.32591991016162	-3.45907916558172	6.37580356010998
H	1.77606551118008	-3.30736291611787	6.98638761298993
H	4.58513371634630	-1.27511560517683	-0.72073997455084

H	-4.06637087178254	-1.00007429250585	1.41911153480596
F	6.12414838193272	-0.24505862795652	1.81539910254427
F	8.67495650179171	-0.73516009083771	1.02160909542590
F	9.41740680613296	-3.26423385459629	0.28810520694052
F	5.03559732581729	-4.81360644546859	1.12155443229259
F	-3.94611170902973	-4.29399759210949	2.03411186713310
F	-6.61610630429572	-4.69449366339528	2.33646449114775
F	-8.04916048152939	-3.12199620042862	4.05543037141423
F	-6.80165131980186	-1.13785015919035	5.46479319741719
F	-4.13604196798295	-0.71951833103363	5.15336047832331
H	-6.76382741922688	-0.61839340898056	-2.19099713131194
H	-1.76022905138083	7.67857424561815	-4.93327403089384
H	0.56216166046969	6.36754067576730	-4.43560628047472
H	-8.05674128336616	3.88294513519687	-4.64298909743186
H	-6.78632891737001	6.25542699688159	-4.99777649742617
H	-4.30102133992098	7.06120985823750	-4.70829632406220
F	1.73054622721478	2.41649082557008	-4.93643640533992
F	4.39797882206269	2.83497142114896	-5.23166571849405
F	5.65281270131167	4.76082358634686	-3.74867625733358
F	4.22862408273797	6.25961772928356	-1.95824706637339
F	1.56605947954117	5.83077505270320	-1.64073667294070
F	-8.58336258566474	1.99892943960281	-1.60008520136702
F	-11.02796703414678	0.98445525520933	-2.21945526800345
F	-11.45251293988899	-0.16158779980096	-4.66609767861771
F	-9.42037482810215	-0.29233612433600	-6.49322934154743
F	-6.97278301132179	0.71569801826639	-5.87605116125166
H	1.70542638533577	2.09233262492992	-1.59249645277824
H	-4.72020467695789	-1.29915989260555	-0.56816485569491
H	2.48940445707325	0.12859905597734	-1.67423532694056

F	7.59236324360238	-5.30070206652687	0.34204068349677

Table S8. Coordinates for triply linked CoDPFPP dimer.

	X	Y	Z
C	-0.87511883805568	-5.16317664174296	5.79929941709485
C	-1.99986029731854	-4.69028429288085	5.19265974517944
C	-1.56630451884507	-3.76653487281595	4.17454523292669
N	-0.18350582968538	-3.66825807652331	4.15484103643928
C	0.24351129833544	-4.52805254675096	5.15534523645235
C	4.78742713942902	-3.61147351145764	4.48715184941885
C	4.00789681969917	-4.38815104837584	5.29070513965461
C	2.64302364471647	-4.14731232383667	4.90531854961443
N	2.57577409976325	-3.22951412823109	3.86818223596345
C	3.89638994126863	-2.89784545238360	3.60702839356530
C	1.55938630719300	-4.75656531698309	5.50685413287870
C	2.75302907787970	0.04628181503147	0.14066057067178
C	3.88775123621906	-0.41242710105986	0.76755364065954
C	3.45678904555936	-1.32846956169710	1.78531443863421
N	2.06914823328582	-1.44548267862379	1.80012019981490
C	1.64240946793242	-0.60480667904279	0.79515743052054
C	4.32539769342609	-2.00499349187846	2.63271196684883
C	-2.89043590416504	-1.49161932114531	1.47042171252873
C	-2.10116669719948	-0.72699139391540	0.64370466730293
C	-0.73684671526428	-0.98449806667310	1.04109160349098
N	-0.67429227703859	-1.88378352102545	2.08324957593520
C	-2.00418352119710	-2.19905921355890	2.35070472135306
C	-2.43473482866631	-3.08116224984160	3.33401155828740
C	0.32977408667836	-0.35843861996271	0.40157436118403
Co	0.94705528820717	-2.55782586275486	2.97814459581147
C	5.78856805716782	-1.76361894347911	2.48419982841187

C	-3.90002332467057	-3.30324373448908	3.49158661239825
C	6.42430279930764	-0.71611313832310	3.15936372866926
C	7.79331799705411	-0.47551502422448	3.02603866901409
C	8.55660570509018	-1.30080405480914	2.19610601654080
C	7.94972274393156	-2.35522028528133	1.50886320687245
C	6.57872826941435	-2.57245751697499	1.66055446269896
C	-4.56143075337361	-4.31917765279343	2.79352949621068
C	-5.93410740363420	-4.53589112748837	2.93060248946058
C	-6.67499277822882	-3.71803825243312	3.78773489261814
C	-6.04217709833477	-2.69477171296559	4.49825823237283
C	-4.66816541337557	-2.50095247698406	4.34214979209248
C	-2.35782591920640	0.18641414762038	-0.43075085640533
C	-3.49313765654674	0.65320425081388	-1.05056738296534
C	-3.06185208678577	1.56369692164658	-2.07317944586229
N	-1.67350240049020	1.67043521545781	-2.09690008765849
C	-1.24662182002227	0.82880878270679	-1.09282520973900
C	3.28508255067721	1.73296220571302	-1.75302189659227
C	2.49645120439666	0.95937218190898	-0.93406528441112
C	1.13276068636664	1.20762816133924	-1.33947351698249
N	1.07024259845434	2.10745521698708	-2.38112912368222
C	2.39932508256828	2.43335943213594	-2.63944342455680
C	0.06627764390015	0.57897257860220	-0.70227637441240
C	1.27369605116966	5.34289007984787	-6.13548907286548
C	2.39683031142080	4.89455125725420	-5.50763448297153
C	1.96249960494145	3.98388093733110	-4.47808903181369
N	0.58067591989947	3.87146224828922	-4.47065990323183
C	0.15503776163145	4.70847450484158	-5.49085447775875
C	2.82980455315964	3.31575505923688	-3.62255705015113
C	-4.39046408400875	3.81312786697684	-4.80403755102066

C	-3.60925714284341	4.56465349771298	-5.62961665283582
C	-2.24441222312928	4.32516859848396	-5.24318834414636
N	-2.17871392914466	3.43019632820611	-4.18622591083973
C	-3.50018355632212	3.11221017929588	-3.91301215516137
C	-3.93036984876316	2.23875852721722	-2.92183923313550
C	-1.15981741702113	4.92196007342823	-5.85541259829869
Co	-0.55062711568874	2.77084879631925	-3.28528722836747
C	4.29411047049608	3.55261535157867	-3.76730005896571
C	-5.39451148722027	2.01257081165335	-2.75951774999590
C	5.08438479469351	2.73696202456984	-4.58422192321392
C	6.45778424117989	2.94467598374706	-4.72712964399918
C	7.06717652855750	3.99604484888371	-4.03741135897844
C	6.30386793405812	4.82793372985935	-3.21411937709175
C	4.93235176424364	4.59683871776080	-3.08964506748956
C	-6.16096504989379	2.80859788521729	-1.90155306364928
C	-7.53240955001196	2.60582197229454	-1.73483677898606
C	-8.16438604331002	1.57954875460029	-2.44178701958293
C	-7.42519794176688	0.76793498612533	-3.30626696665247
C	-6.05504318979641	0.99358267889893	-3.45390750076455
H	-0.79160738038562	-5.87769496950483	6.61328541208056
H	-3.03550822791188	-4.93622649946853	5.40412087303964
H	5.86880942433298	-3.52042066047823	4.47889567873392
H	4.31281938756144	-5.06849085111384	6.08076798338144
H	1.75554308783950	-5.46107014616111	6.31453139444583
H	4.92192536333364	-0.15745100028053	0.55992481041363
H	-3.97260022919631	-1.57157843226623	1.48405219678588
F	5.71044117940839	0.09060879506952	3.96349837042509
F	8.38051647557638	0.53589472336710	3.68395039015269
F	9.87097030275942	-1.08084607157286	2.05910792719876

F	8.68649776747897	-3.14497240216735	0.71263606877113
F	6.01618973890844	-3.59128598078202	0.98777080909740
F	-3.86966490692507	-5.11879199239783	1.96340757609380
F	-6.54543022151099	-5.51845664459154	2.25131799808506
F	-7.99250885285189	-3.91551042769171	3.92871218438866
F	-6.75758891614304	-1.91106065169813	5.31956739732912
F	-4.08139305097977	-1.50980846048238	5.03524177901774
H	-4.52777126566514	0.40500285495598	-0.83708281072261
H	1.19123224011628	6.04128712079456	-6.96345350817979
H	3.43194308564930	5.14769165484457	-5.71310789468557
H	-5.47244733399533	3.73050942302181	-4.78828497648260
H	-3.91311590227891	5.22875007806890	-6.43379033607779
H	-1.35458825600867	5.60879686997989	-6.67851002481385
F	4.51984350696270	1.71983552097917	-5.25793929174535
F	7.19450995588037	2.14840662002996	-5.51694822949653
F	8.38390391880534	4.20692275098837	-4.16583688190139
F	4.21882382592975	5.40930154282590	-2.29103738652362
F	-5.57478975727501	3.80182780377584	-1.21096352492664
F	-8.24617324778806	3.38381770842314	-0.90666431345194
F	-9.47942795334634	1.37348577874433	-2.29052352355330
F	-8.03552171513587	-0.21765264217020	-3.98209863352365
F	-5.36463040268000	0.19861707835640	-4.28967844951044
H	4.36658834746980	1.82216773502658	-1.75857985385881
F	6.89349601971716	5.83640705738853	-2.55409584434294

References

(1) Bengasi, G.; Baba, K.; Frache, G.; Desport, J.; Gratia, P.; Heinze, K.; Boscher, N. D. Conductive Fused Porphyrin Tapes on Sensitive Substrates by a Chemical Vapor Deposition Approach. *Angewandte Chemie International Edition* **2019**, *58* (7), 2103-2108. DOI: https://doi.org/10.1002/anie.201814034.
(2) Bengasi, G.; Baba, K.; Back, O.; Frache, G.; Heinze, K.; Boscher, N. D. Reactivity of Nickel(II) Porphyrins in oCVD Processes—Polymerisation, Intramolecular Cyclisation and Chlorination. *Chemistry – A European Journal* **2019**, *25* (35), 8313-8320. DOI: https://doi.org/10.1002/chem.201900793.
(3) Baba, K.; Bengasi, G.; El Assad, D.; Grysan, P.; Lentzen, E.; Heinze, K.; Frache, G.; Boscher, N. D. Conductive Directly Fused Poly(Porphyrin) Coatings by Oxidative Chemical Vapour Deposition – From Single- to Triple-Fused. *European Journal of Organic Chemistry* **2019**, *2019* (13), 2368-2375. DOI: https://doi.org/10.1002/ejoc.201900045.
(4) Bengasi, G.; Quétu, L.; Baba, K.; Ost, A.; Cosas Fernandes, J. P.; Grysan, P.; Heinze, K.; Boscher, N. D. Constitution and Conductivity of Metalloporphyrin Tapes. *European Journal of Inorganic Chemistry* **2020**, *2020* (20), 1938-1945. DOI: https://doi.org/10.1002/ejic.202000243.
(5) Audinot, J. N.; Philipp, P.; De Castro, O.; Biesemeier, A.; Hoang, Q. H.; Wirtz, T. Highest resolution chemical imaging based on secondary ion mass spectrometry performed on the helium ion microscope. *Rep Prog Phys* **2021**, *84* (10). DOI: 10.1088/1361-6633/ac1e32 From NLM.
(6) Neese, F. The ORCA program system. *WIREs Computational Molecular Science* **2012**, *2* (1), 73-78. DOI: https://doi.org/10.1002/wcms.81.
(7) Neese, F. Software update: the ORCA program system, version 4.0. *WIREs Computational Molecular Science* **2018**, *8* (1), e1327. DOI: https://doi.org/10.1002/wcms.1327.
(8) Neese, F.; Wennmohs, F.; Becker, U.; Riplinger, C. The ORCA quantum chemistry program package. *The Journal of Chemical Physics* **2020**, *152* (22), 224108. DOI: 10.1063/5.0004608.
(9) Becke, A. D. Density-functional exchange-energy approximation with correct asymptotic behavior. *Physical Review A* **1988**, *38* (6), 3098-3100. DOI: 10.1103/PhysRevA.38.3098.
(10) Perdew, J. P. Density-functional approximation for the correlation energy of the inhomogeneous electron gas. *Physical Review B* **1986**, *33* (12), 8822-8824. DOI: 10.1103/PhysRevB.33.8822.
(11) Weigend, F.; Ahlrichs, R. Balanced basis sets of split valence, triple zeta valence and quadruple zeta valence quality for H to Rn: Design and assessment of accuracy. *Physical Chemistry Chemical Physics* **2005**, *7* (18), 3297-3305, 10.1039/B508541A. DOI: 10.1039/B508541A.
(12) Schäfer, A.; Horn, H.; Ahlrichs, R. Fully optimized contracted Gaussian basis sets for atoms Li to Kr. *The Journal of Chemical Physics* **1992**, *97* (4), 2571-2577. DOI: 10.1063/1.463096.
(13) Schäfer, A.; Huber, C.; Ahlrichs, R. Fully optimized contracted Gaussian basis sets of triple zeta valence quality for atoms Li to Kr. *The Journal of Chemical Physics* **1994**, *100* (8), 5829-5835. DOI: 10.1063/1.467146.
(14) Weigend, F. Accurate Coulomb-fitting basis sets for H to Rn. *Physical Chemistry Chemical Physics* **2006**, *8* (9), 1057-1065, 10.1039/B515623H. DOI: 10.1039/B515623H.
(15) Grimme, S.; Antony, J.; Ehrlich, S.; Krieg, H. A consistent and accurate ab initio parametrization of density functional dispersion correction (DFT-D) for the 94 elements H-Pu. *The Journal of Chemical Physics* **2010**, *132* (15), 154104. DOI: 10.1063/1.3382344.

(16) Grimme, S.; Ehrlich, S.; Goerigk, L. Effect of the damping function in dispersion corrected density functional theory. *Journal of Computational Chemistry* **2011**, *32* (7), 1456-1465. DOI: https://doi.org/10.1002/jcc.21759.
(17) Neese, F.; Wennmohs, F.; Hansen, A.; Becker, U. Efficient, approximate and parallel Hartree-Fock and hybrid DFT calculations. A 'chain-of-spheres' algorithm for the Hartree-Fock exchange. *Chemical Physics* **2009**, *356*, 98-109. DOI: 10.1016/j.chemphys.2008.10.036.
(18) Izsák, R.; Neese, F. An overlap fitted chain of spheres exchange method. *The Journal of Chemical Physics* **2011**, *135* (14), 144105. DOI: 10.1063/1.3646921.
